# Effects of drought and salt-stresses on gene expression in *Caragana korshinskii* seedlings revealed by RNA-seq

**DOI:** 10.1186/s12864-016-2562-0

**Published:** 2016-03-08

**Authors:** Shaofeng Li, Chengming Fan, Yan Li, Jianhui Zhang, Jingshuang Sun, Yuhong Chen, Changyan Tian, Xiaohua Su, Mengzhu Lu, Chengzhi Liang, Zanmin Hu

**Affiliations:** Experimental Center of Forestry in North China, Chinese Academy of Forestry, Beijing, 100023 P. R. China; Institute of Genetics and Developmental Biology, Chinese Academy of Sciences, Datun Road, Chaoyang District Beijing, 100101 China; Xinjiang Institute of Ecology and Geography, Chinese Academy of Sciences, Urumqi, 830011 P. R. China; State Key Laboratory of Tree Genetics and Breeding, Research Institute of Forestry, Chinese Academy of Forestry, Beijing, 100091 P. R. China

**Keywords:** *Caragana korshinskii*, Transcriptome, Illumina sequencing, Drought-stress tolerance, Salt-stress tolerance

## Abstract

**Background:**

Drought and soil salinity are major abiotic stresses. The mechanisms of stress tolerance have been studied extensively in model plants. *Caragana korshinskii* is characterized by high drought and salt tolerance in northwestern China; unique patterns of gene expression allow it to tolerate the stress imposed by dehydration and semi-desert saline soil. There have, however, been no reports on the differences between *C. korshinskii* and model plants in the mechanisms underlying their drought and salt tolerance and regulation of gene expression.

**Results:**

Three sequencing libraries from drought and salt-treated whole-seedling- plants and the control were sequenced to investigate changes in the *C. korshinskii* transcriptome in response to drought and salt stresses. Of the 129,451 contigs, 70,662 (54.12 %) were annotated with gene descriptions, gene ontology (GO) terms, and metabolic pathways, with a cut-off E-value of 10^−5^. These annotations included 56 GO terms, 148 Kyoto Encyclopedia of Genes and Genomes (KEGG) pathways, and 25 Clusters of Orthologous Groups (COG). On comparison of the transcriptomes of the control, drought- and salt-treated plants, 1630 and 1521 contigs showed significant differences in transcript abundance under drought and salt stresses. Compared to the differentially expressed genes (DEGs) in drought- or salt-treated *Arabidopsis* in the database, 542 DEGs in drought-treated *C. korshinskii* and 529 DEGs in salt-treated samples were presumably unique to *C. korshinskii*. The transcription profiles revealed that genes related to transcription factors, protein kinases, and antioxidant enzymes are relevant to the tolerance of drought and salt stress in this species. The expression patterns of 38 randomly selected DEGs were confirmed by quantitative real-time PCR and were essentially consistent with the changes in transcript abundance identified by RNA-seq.

**Conclusions:**

The present study identified potential genes involved in drought and salt tolerance in *C. korshinskii*, as well as many DEGs uniquely expressed in drought- or salt-treated *C. korshinskii* samples compared to *Arabidopsis*. To our knowledge, this study is the first exploration of the *C. korshinskii* transcriptome under drought and salt conditions, and these results will facilitate the discovery of specific stress-resistance-related genes in *C. korshinskii*, possibly leading to the development of novel plant cultivars through genetic engineering.

**Electronic supplementary material:**

The online version of this article (doi:10.1186/s12864-016-2562-0) contains supplementary material, which is available to authorized users.

## Background

Environmental stresses or abiotic constraints such as salinity, drought, heavy metals and low temperature are major obstacles to plant survival and development in many places worldwide. Plant responses and adaptations to abiotic stresses are complex [1]. Many cellular and physiological processes such as signal perception and transduction [2, 3], regulation of transcription, membrane trafficking, energy metabolism [4], and protein phosphorylation and dephosphorylation are involved in plants’ toleration of excessive salt and drought stresses. Several studies on drought and salt stresses using microarrays, transcriptome sequencing, and microRNA sequencing [5–7] have identified stress-inducible genes involved in water transport (aquaporins), ion transport (plasma membrane (PM) H^+^-ATPase, Na^+^/H^+^ exchanger or Na^+^/H^+^ antiporter), cellular membrane integrity (proline, glycine betaine, mannitol), scavenging of free oxygen radicals (superoxide dismutase, catalase and peroxidase), and protecting macromolecules (late embryogenesis abundant proteins and chaperones). Other proteins, such as regulatory proteins (transcription factors, protein kinases, protein phosphatases, and calmodulin-binding proteins), were found to be involved in signal transduction [8, 9]. ABA-dependent and ABA-independent signaling pathways were induced by plants in response to stress [10]. The ABA-dependent transcription factors include MYC/MYB and ABA-responsive element binding/ABA-binding factor (AREB/ABF). The ABA-independent transcription factors include dehydration-responsive element-binding proteins (DREB), C-repeat/drought-responsive element (CRT/DRE), and CRT/DRE-binding factor (CBF) [11, 12]. Other transcription factors responding to abiotic stress conditions are zinc-finger proteins [13], basic-domain leucine-zipper (bZIP) [14], WRKY [15], and NACs [16].

The development of next-generation sequencing technology (NGS) has led to major progress in understanding plant responses to drought and salt stress [17–19]. Some recent efforts include tissue-selective signaling and hormone crosstalk in response to salt and osmotic stresses and, recently, the dynamics of transcriptional networks that are functional at the seedling stage in response to water stress in *Gossypium arboreum* [20]. The transcriptional characteristics of genes related to the reactive oxygen species (ROS)-scavenging system are important to the salt tolerance of *Reaumuria trigyna* [21]. Further, genome-wide gene-expression profiling was used to examine the molecular mechanism and physiological response of *Gossypium herbaceum* to drought [22]. Thumma et al. [23] used RNA sequencing (RNA-seq) to study the effect of water stress on gene expression in *Eucalyptus camaldulensis* seedlings derived from three natural populations. RNA-seq technology and its applications have dramatically accelerated plant-genomics research, including high-throughput sequencing of non-model-plant transcriptomes and large-scale genome-wide expression analysis.

*Caragana korshinskii* is a leguminous shrub with highly developed root systems and strong stress tolerance [24, 25] that is commonly found in arid and semi-arid lands in northwestern China and Mongolia [26, 27]. Therefore, *C. korshinskii* is widely planted in China to prevent desertification and improve vegetation coverage. The effect of drought or salt stress on plant gene expression has been intensely studied in numerous species including *Arabidopsis* [14], rice [28], maize [15], wheat (*Triticum aestivum* L.) [29], and *Sorghum bicolor* [30]. In *C. korshinskii*, some investigations have focused on growth properties, nutritive characteristics, evapotranspiration, and genetic diversity [31–33], and several drought- and salt-stress-response genes have already been characterized [34–36], but there have been no studies on the stress-signaling pathways. This lack of molecular studies is a significant obstacle to understanding the molecular mechanisms underlying drought or salt adaptation in *C. korshinskii*.

In this study, we generated transcriptome datasets to explore the salt- and drought-tolerance mechanisms of *C. korshinskii* using the Illumina HiSeq™ 2000 platform. The main objectives of this study are to identify genes showing transcriptional differences and to identify their putative functions, as well as to identify genes putatively unique to *C. korshinskii* compared to the model plant *Arabidopsis* and describe the patterns in their transcript abundance under salt- and drought-treated conditions. The assembled, annotated transcriptome data and differential expression profiles will facilitate further genetic and genomics studies on the molecular mechanisms of salt and drought tolerance in *C. korshinskii* and most likely in other leguminous plants.

## Results

### Illumina sequencing and *de novo* assembly

In total, 120.26 million raw reads were generated from control samples, 39.78 million raw reads were generated from drought-treated samples, and 39.90 million raw reads were generated from salt-treated samples. We obtained a total of 6.66 gigabase pairs (Gbp) with an average GC content of 47.66 %. We obtained approximately 199.94 million total reads, of which approximately 182.93 million passed the Illumina quality filtering, yielding a quality rate of over 91.49 %. This result indicated that the read number and quality were high enough for further analysis (the number of clean reads for each sample is shown in Additional file 1). The 182.93 million high-quality reads were assembled into 129,451 contigs with an N50 length of 1332 bp and an N90 length of 283 bp (Additional file 1, Table 1). All 129,451 contigs were longer than 100 bp; 99,528 contigs (76.78 %) ranged from 200 to 1000 bp; 19,477 contigs (15.03 %) ranged from 1001 to 2000 bp; and 10,622 contigs (8.19 %) were longer than 2 kb, among which 2.63 % (3415) contigs were more than 3000 bp long (Table 1, Additional file 2). The RNA-seq data can be found in the National Center for Biotechnology Information (NCBI) Sequence Read Archive (SRA) database under number SRP061143.

**Table 1:** Summary of *Caragana korshinskii* transcriptome assembly in this study compared with the study by Long et al. [38]

Category	Number				Total number	Mean length (bp)	N50 (bp)
	200–500 bp	500–1kbp	1 k–2kbp	>2kbp			
Contig	74,830	24,698	19,477	7207	129,451	758.70	1332
Unigene	54,117	15,417	10,235	6496	86,265	709	1231

Contig showed the transcriptome assembly in this study

Unigene showed the transcriptome assembly by long et al. [38]

### Functional annotation and analysis

Among the 129,451 transcripts, the direction could be determined for 70,662. Altogether, 70,491 (54.45 %) contigs were successfully annotated in the nr, GO, KEGG, COG, Swiss-Prot and InterProScan databases. These contigs are listed in Additional file 3. Of these, 70,062 (54.12 %) of 129,451 contigs had significant matches in the nr database, 62,605 (43.36 %) contigs aligned to the KEGG database, 47,462 (36.66 %) contigs matched GO annotations, and 8321 (6.43 %) contigs were similar to proteins in the InterProScan database (Additional file 4). Because of the lack of genome and EST information for *C. korshinskii*, 45.55 % (58,960) of the contigs did not match any known genes in the database (Additional file 5).

**Fig. 1:**
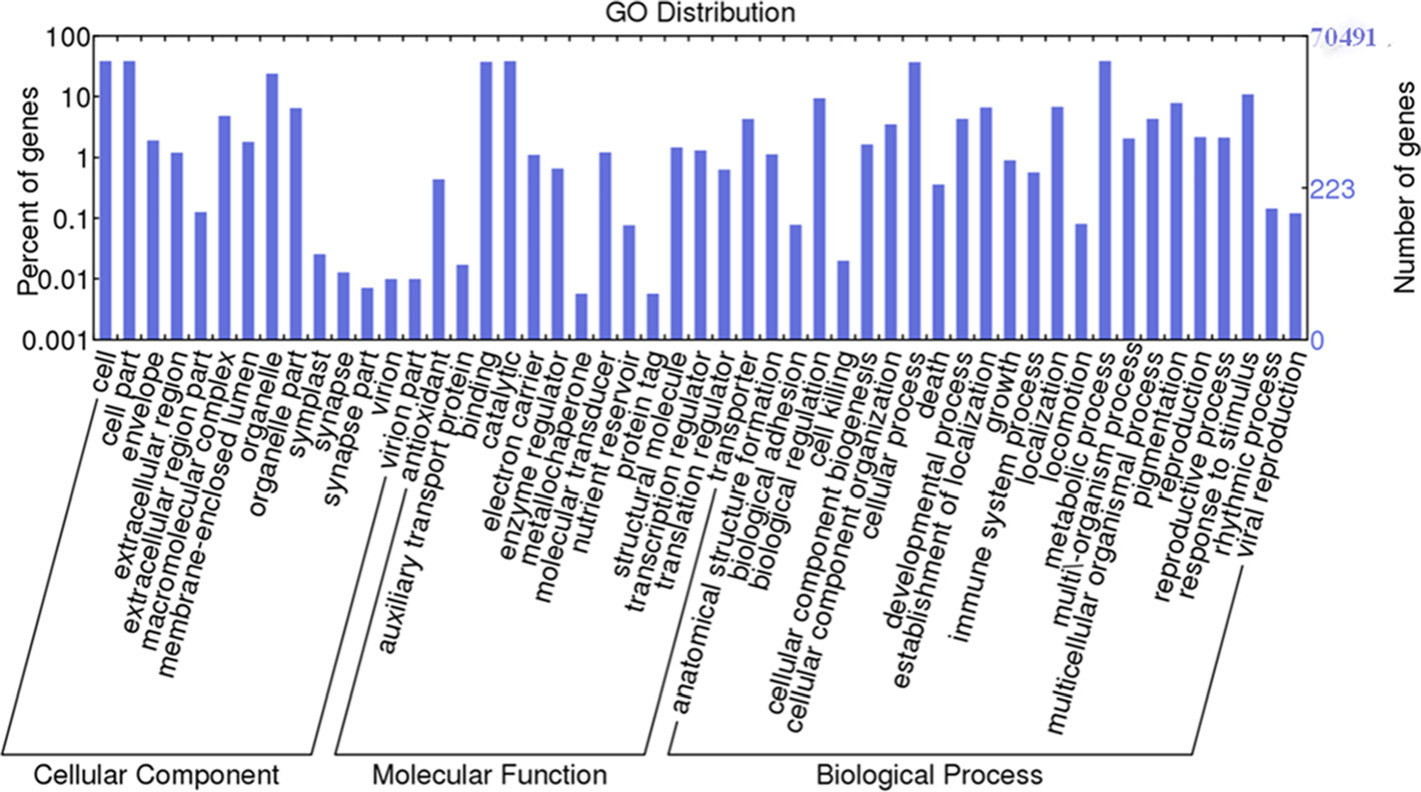
Histogram of gene ontology (GO) classification. Contigs with the best BLAST hits were aligned to the GO database. A total of 53,950 (41.62 %), 46,782 (22.29 %), and 28,894 (22.29 %) contigs of *C. korshinskii* were classified into 56 terms from three ontologies involving cellular components, biological processes, and molecular function. The right Y-axis indicates number of contigs in a category. The left Y-axis represents the percentage of a specific category of contigs in the main category

**Fig. 2:**
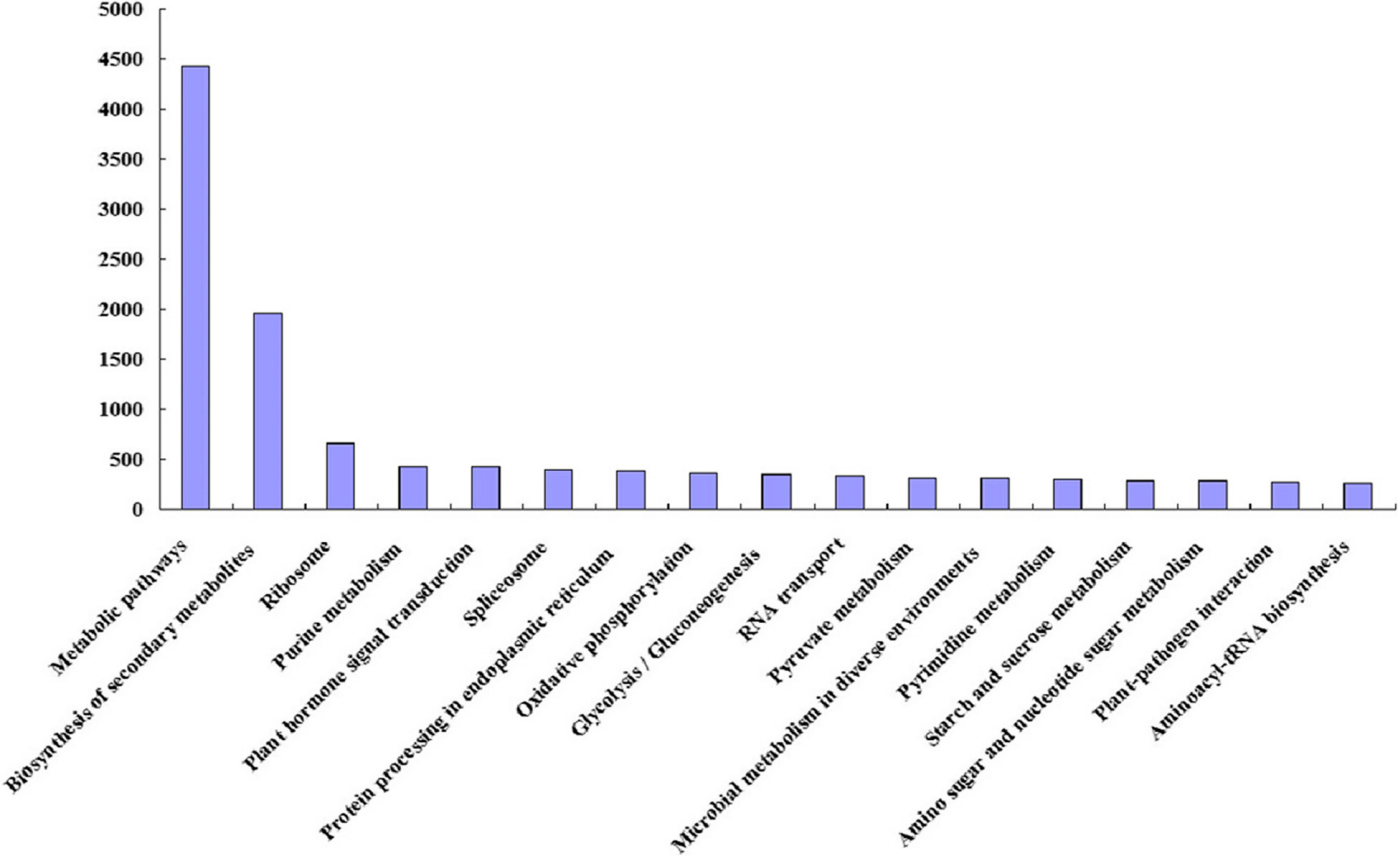
KEGG-enrichment analysis in *C. korshinskii*. The Y-axis indicates the numbers of contigs enriched in KEGG pathways. The X-axis represents contigs enriched in different metabolic pathways

After Blast2GO analysis, the contigs were classified into 56 terms from three ontologies involved in cellular components, biological processes, and molecular function (Fig. 1). In each of the three main GO classifications (biological process, cellular component, and molecular function), “metabolic process”, “cell” or “cell part”, and “catalytic activity” were dominant among the returned terms. Based on a comparison to the KEGG database, 21,104 (39.57 %) of the 70,491 annotated contigs had significant matches and were thus assigned to 148 KEGG pathways. The most-represented pathways were “metabolic pathways” (4425 contigs), “biosynthesis of secondary metabolites” (1967 contigs), “ribosome” (660 contigs), “purine metabolism” (425 contigs), and “plant hormone signal transduction” (423 contigs). A total of 94,820 contigs showed a COG classification. Among the 25 COG categories, the cluster for “General function prediction only” (30,370, 32.03 %) was the largest, followed by “Posttranslational modification, protein turnover, chaperones” (7851, 8.28 %), “Signal transduction mechanisms” (7739, 8.16 %), “Carbohydrate transport and metabolism” (4411, 4.65 %), and “Transcription” (4163, 4.39 %). The categories “Cell motility” (44, 0.000464 %), and “Nuclear structure” (244, 0.26 %) had the fewest corresponding genes (Fig. 2, Additional file 6).

### Analysis of differential expression of assembled *C. korshinskii* transcripts under drought and salt treatments

**Fig. 3:**
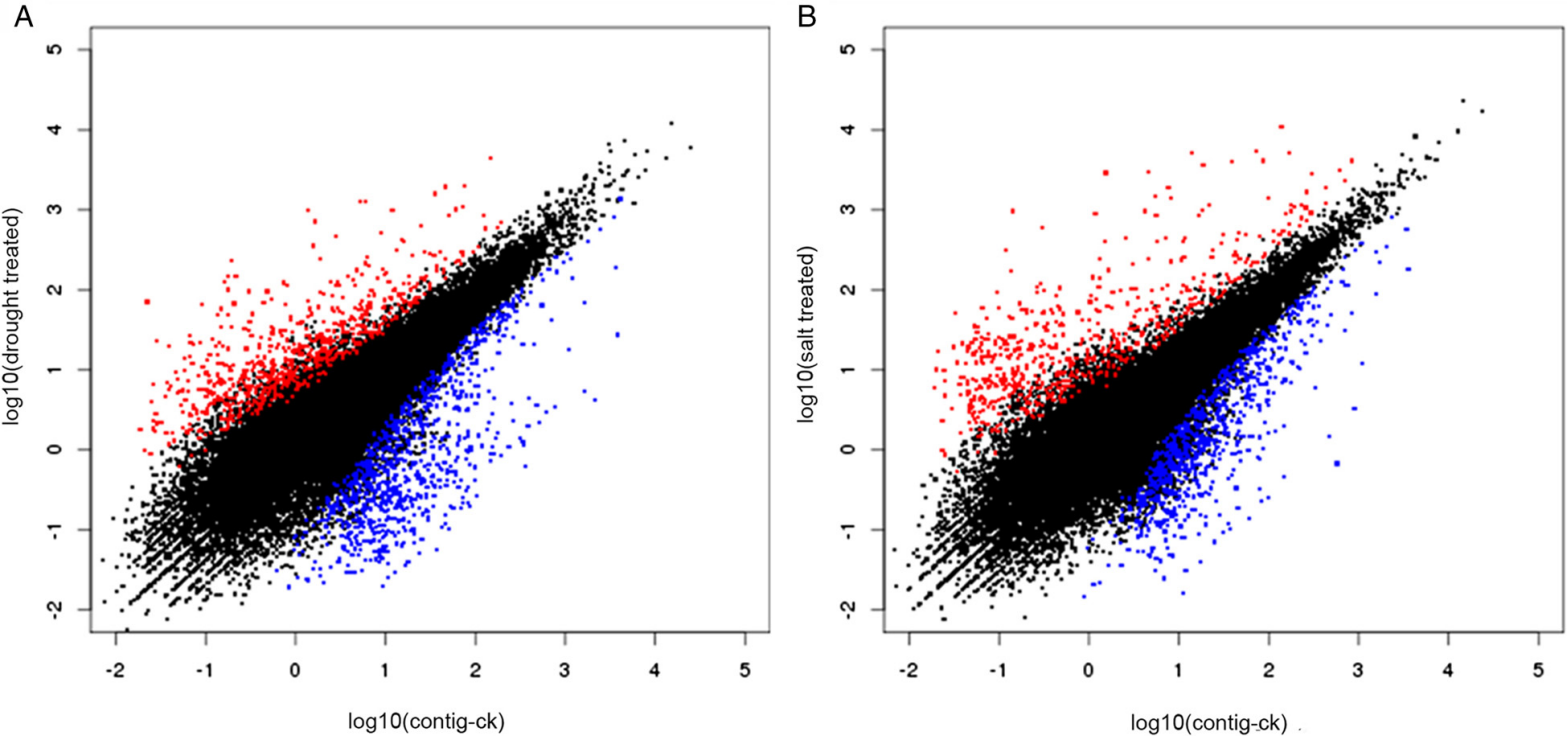
Identification of DEGs between drought- (**a**) and salt- (**b**) treated *C. korshinskii*. DEGs were filtered using FDR ≤0.05 and the absolute value of log_2_Ratio ≥1 as the threshold. *Red spots* represent upregulated DEGs, and *blue spots* indicate downregulated DEGs. *Black spots* represent contigs that did not show obvious changes in drought- or salt-treated *C. korshinskii*

**Fig. 4:**
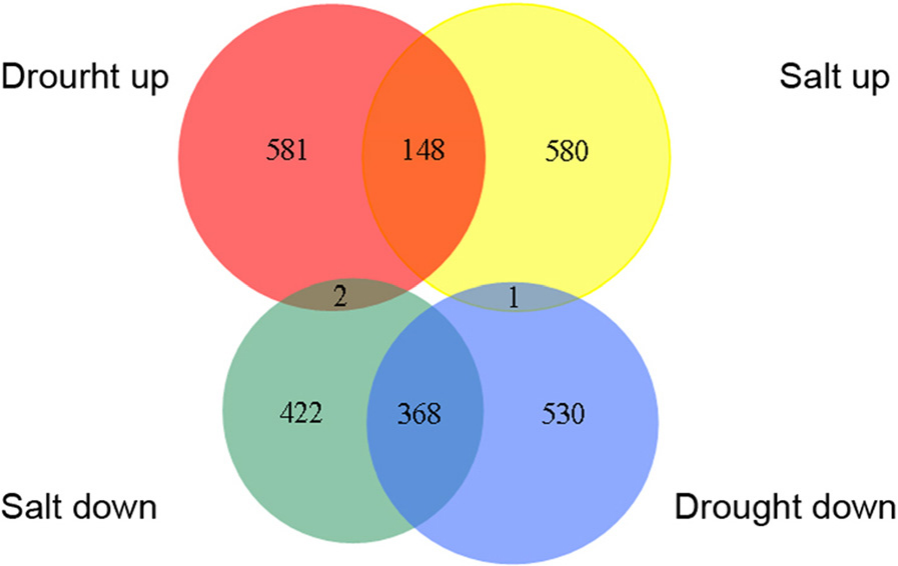
Venn diagrams illustrate the DEGs under drought and salt treatment in *C. korshinskii*. The *red* and *blue colors* represent the upregulated and downregulated transcripts under drought treatment, respectively. The *yellow* and *green colors* represent the upregulated and downregulated transcripts under salt treatment, respectively

Compared with the control, 1630 and 1521 contigs were classified as differentially expressed genes (DEGs) in drought- and salt-treated *C. korshinskii*, respectively (Additional file 7). In the drought-treated seedlings, 731 contigs showed increased transcript abundance and 899 contigs showed decreased transcript abundance. In the salt-treated seedlings, 729 transcripts were upregulated and 792 transcripts were downregulated (Figs. 3 and 4).

Venn-diagram analysis revealed the specificity and overlap among differentially expressed genes (Fig. 4). Among 1630 and 1521 differentially expressed genes, numerous genes overlapped in expression in response to the salt and drought treatments. For example, in drought- and salt-treated plants, 148 upregulated transcripts overlapped and 368 downregulated transcripts overlapped. In addition, 1005 of 1521 contigs appeared only in salt-treated samples, while 1111 of 1630 contigs were expressed only in drought-treated samples.

GO annotation and statistical analyses demonstrated that 1844 tested DEGs (some contigs have more than one GO annotation) were classified into three GO ontologies and 60 terms in drought-treated plants. Of these DEGs, 843 were associated with biological processes, 419 with cellular components, and 612 with molecular function. Among these GO categories, “oxidoreductase activity” (GO:0016491,168 DEGs), “oxidation-reduction process” (GO:0055114,146 DEGs), and “response to stress” (GO:0006950, 149 DEGs) were significantly enriched among DEGs compared to the whole transcriptome background. In the molecular-function category, 2640 tested DEGs (some contigs have more than one GO annotation) were enriched in 63 terms in salt-treated samples. The overwhelming majority of the genes with differential expression between the control and salt-treated conditions were also related to “oxidoreductase activity” (GO:0016491,186 DEGs), “oxidation-reduction process” (GO:0055114,169 DEGs), and “response to stress” (GO:0006950,131 DEGs). Approximately 41.7 % of 60 GO terms in drought-treated *C. korshinskii* were also found in salt-treated *C. korshinskii*, such as oxidoreductase activity, oxidation-reduction process, electron carrier activity, and monooxygenase activity (Additional file 8).

**Fig. 5:**
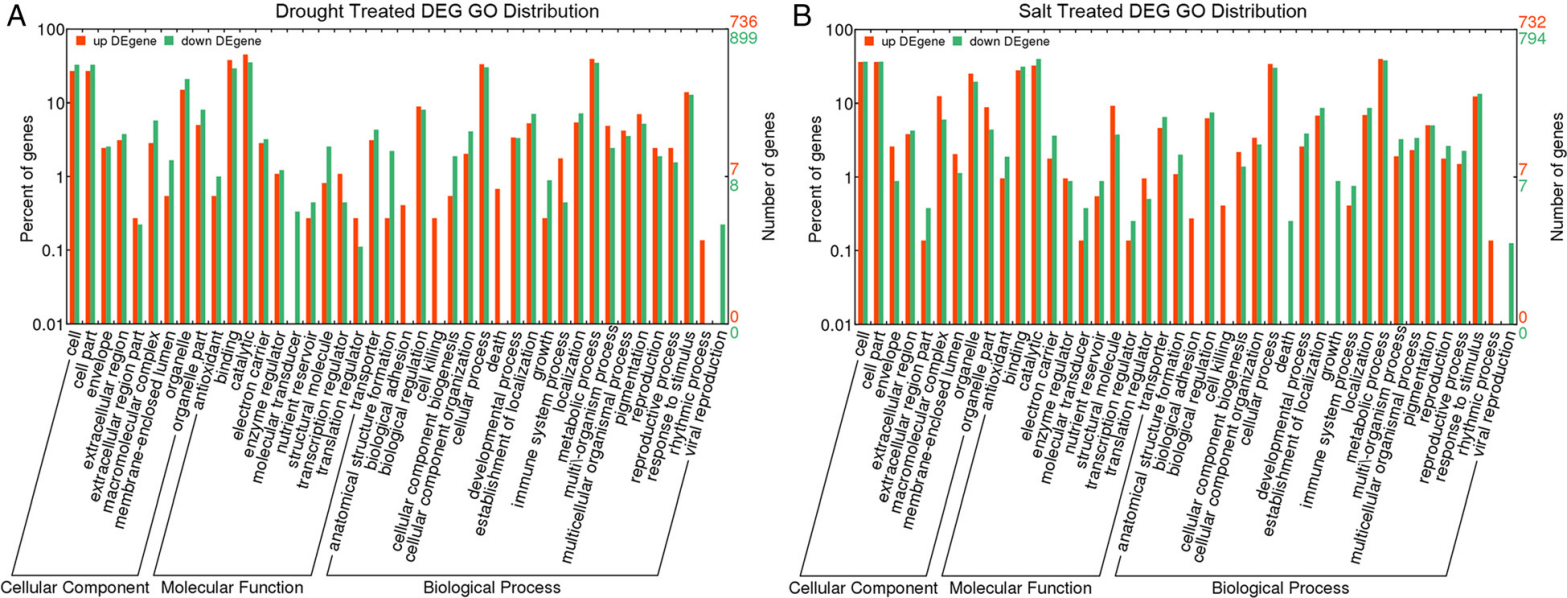
GO categories of the DEGs in drought- or salt-treated *C. korshinskii*. DEGs with the best BLAST hits were aligned to the GO database. Each bar represents the percentage and number of DEGs mapped to each GO category. The color indicates DEGs that were upregulated or downregulated in drought- (**a**) or salt- (**b**) treated *C. korshinskii*: *red* for upregulated DEGs and *green* for downregulated DEGs. The right Y-axis indicates number of contigs in a category. The left Y-axis represents the percentage of a specific category of contigs in the main category

A total of 731 DEGs that were significantly upregulated in drought-treated *C. korshinskii* were categorized into 46 GO terms based on sequence similarity. Accordingly, 899 significantly downregulated DEGs were categorized into 61 GO terms (Fig. 5a, Additional file 9). In addition, 6 GO terms were found in two samples, including extracellular region, monooxygenase activity, serine-type endopeptidase inhibitor activity, iron ion binding, electron carrier activity and heme binding. Interestingly, 25 DEGs were classified into the category “iron ion binding” and 21 DEGs into the category “electron carrier activity” out of 731 upregulated DEGs in drought-treated *C. korshinskii*. Another 22 DEGs were classified into the category “iron ion binding” and 27 DEGs into the “electron carrier activity” among 899 downregulated DEGs in drought-treated *C. korshinskii* (Additional file 9). A total of 729 DEGs that were upregulated in salt-treated *C. korshinskii* were assigned to GO classes with 19 functional terms. Accordingly, 792 DEGs that were significantly downregulated were categorized into 72 GO terms (Fig. 5b, Additional file 9). Generally, 6 GO terms, such as structural constituent of ribosome, extracellular region, ribosome, translation, heme binding, and oxidation-reduction process, were found in both samples. Furthermore, 56 DEGs were classified into the category “oxidation-reduction process” in 729 upregulated in salt-treated *C. korshinskii*, and 95 DEGs were classified into the category “oxidation-reduction process”, out of 792 DEGs in salt-treated *C. korshinskii*, which were are valuable for studying salt-treated *C. korshinskii* (Additional file 9).

In our analysis, 1603 tested DEGs from drought-treated samples mapped to 12 pathways (Corrected *P*-Value <0.05), with most mapping to “metabolic pathways” (28 DEGs), “biosynthesis of secondary metabolites” (18 DEGs), “porphyrin and chlorophyll metabolism” (6 DEGs), “starch and sucrose metabolism” (6 DEGs), and “cysteine and methionine metabolism” (5 DEGs). In salt-treated samples, 1011 DEGs were enriched in 15 metabolic pathways. The most-enriched metabolic pathways were “ribosome” (53 DEGs), “metabolic pathways” (25 DEGs), “biosynthesis of secondary metabolites” (17 DEGs), “glycolysis/gluconeogenesis” (10 DEGs), and “phenylpropanoid biosynthesis” (10 DEGs). Among these metabolic pathways, starch and sucrose metabolism, biosynthesis of secondary metabolites, metabolic pathways, pentose and glucuronate interconversions, linoleic-acid metabolism, and the ribosome were enriched in both drought- and salt-treated *C. korshinskii* (Additional file 10).

**Fig. 6:**
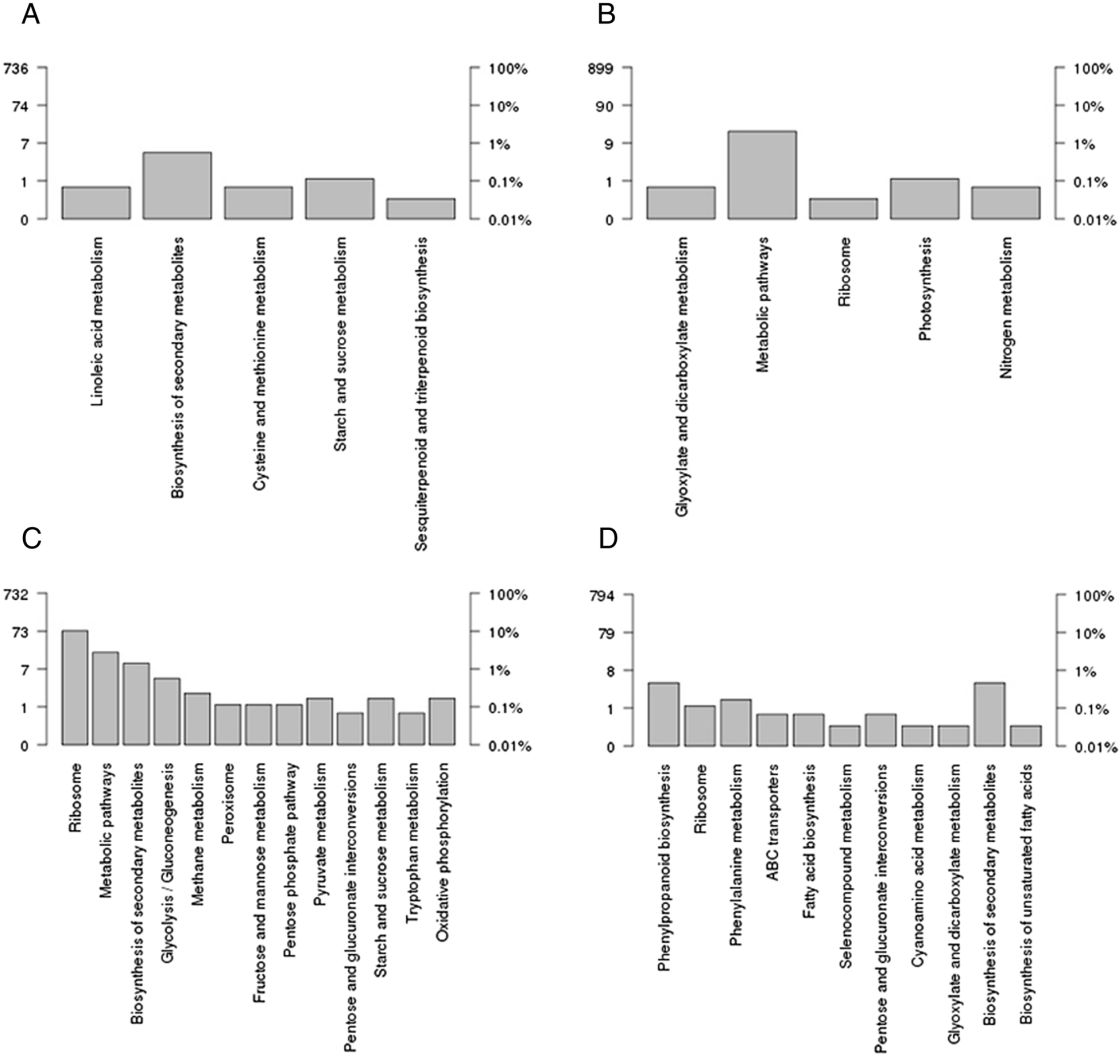
KEGG pathways analysis of the DEGs in drought- or salt-treated *C. korshinskii*. In drought-treated *C. korshinskii*, 731 upregulated DEGs were enriched in KEGG pathways (**a**), and 899 downregulated DEGs were enriched in metabolic pathways (**b**). In salt-treated *C. korshinskii*, 729 upregulated DEGs were mapped to KEGG categories (**c**), and 792 downregulated DEGs were assigned to KEGG pathways (**d**). The left Y-axis indicates the number of DEGs in a KEGG pathway. The right Y-axis represents the percentage of a specific category of DEGs in the pathway

In drought-treated *C. korshinskii*, 731 upregulated DEGs were aligned with the KEGG database and were assigned to 5 KEGG pathways (Fig. 6a, Additional file 11). The most enriched metabolic pathways were “Biosynthesis of secondary metabolites” (456 DEGs), “Starch and sucrose metabolism” (84 DEGs) and “Cysteine and methionine metabolism” (38 DEGs). In addition, 899 downregulated DEGs were enriched in 6 metabolic pathways (Fig. 6b, Additional file 11). Most of these unigenes were sorted to “Metabolic pathways” (869 DEGs), “Photosynthesis” (43 DEGs) and “Porphyrin and chlorophyll metabolism” (25 DEGs) (Additional file 11). In salt-treated *C. korshinskii*, 729 upregulated DEGs mapped to 13 categories of KEGG (Fig. 6c, Additional file 11), including “Metabolic pathways” (378 DEGs), “Biosynthesis of secondary metabolites” (174 DEGs) and “Ribosome” (70 DEGs). Interestingly, 18 DEGs were assigned to the “Peroxisome” pathway. Meanwhile, 792 downregulated DEGs were assigned to 11 KEGG pathways (Fig. 6d, Additional file 11). The pathways with the highest number of DEGs were “Biosynthesis of secondary metabolites” (380 DEGs), “Phenylpropanoid biosynthesis” (63 DEGs) and “Phenylalanine metabolism” (51 DEGs). The candidate functional genes that were differentially expressed in drought- or salt-treated *C. korshinskii* were analyzed (Additional file 12). Many genes that were upregulated or downregulated in response to drought or salt stress in *C. korshinskii* were reported to be involved in multiple mechanisms that might contribute to drought or salt tolerance. Among those functional genes, 171 contigs were upregulated and 169 contigs were downregulated in drought-treated *C. korshinskii*, whereas 98 contigs were upregulated and 189 contigs were downregulated in salt-treated *C. korshinskii*.

### Functional genes related to protein kinase

**Fig. 7:**
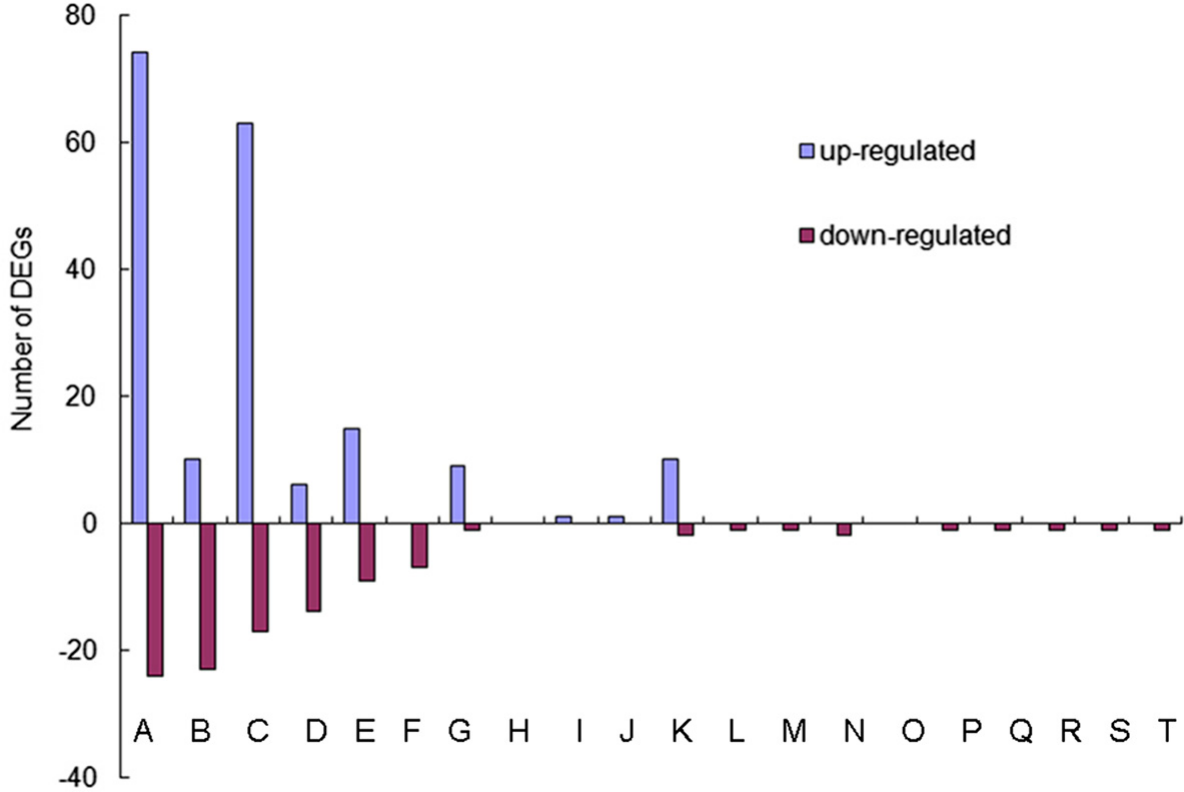
Transcriptional characteristics of DEGs related to protein kinase. The 20 points (A - T) from left to right on the X-axis represent contigs encoding protein kinase under drought (A) or salt stress (B), contigs encoding receptor-like protein kinase under drought (C) or salt stress (D), contigs encoding leucine-rich-repeat receptor-like protein kinase under drought (E) or salt stress (F), contigs encoding lectin receptor-like kinase under drought (G) or salt stress (H), contigs encoding mitogen-activated protein kinase under drought (I) or salt stress (J), contigs encoding cysteine-rich receptor-like protein kinase under drought (K) or salt stress (L), contigs encoding proline-rich receptor-like protein kinase under drought (M) or salt stress (N), contigs encoding tyrosine-protein kinase-like under drought (O) or salt stress (P), contigs encoding protein kinase C under drought (Q) or salt stress (R), and contigs encoding ribosomal protein S6 kinase under drought (S) or salt stress (T). Blue bar represents upregulated DEGs; red bar indicates downregulated DEGs

We identified 131 differentially expressed transcripts predicted to encode protein kinases (Fig. 7, Additional file 13), of which 80 and 20 contigs encoding receptor-like protein kinase were differentially expressed in drought-treated and salt-treated libraries, respectively. Among these receptor-like protein kinases, 24 and seven contigs predicted to encode leucine-rich repeat (LRR) receptor-like protein kinase were differentially expressed in response to drought stress and salt stress. Among these contigs, Cko_contig_70567, Cko_contig_105960 and Cko_contig_30368 were upregulated more than 32-fold under drought stress.

We further identified 289 contigs responsible for mitogen-activated protein kinases, of which two contigs (Cko_contig_220 and Cko_contig_10124) were upregulated dramatically by drought or salt stress (Additional file 14). In addition, eight G-type lectin S-receptor-like serine/threonine-protein kinases were identified. Among these kinases, three (Cko_contig_99573, Cko_contig_39198, and Cko_contig_36276) were upregulated more than 32-fold under drought stress (Additional file 13). We also found that 12 cysteine-rich repeat receptor-like protein kinase (CRK) genes showed differential expression (Additional file 13).

### Transcription factors responding to drought and salt treatments in *C. korshinskii*

**Fig. 8:**
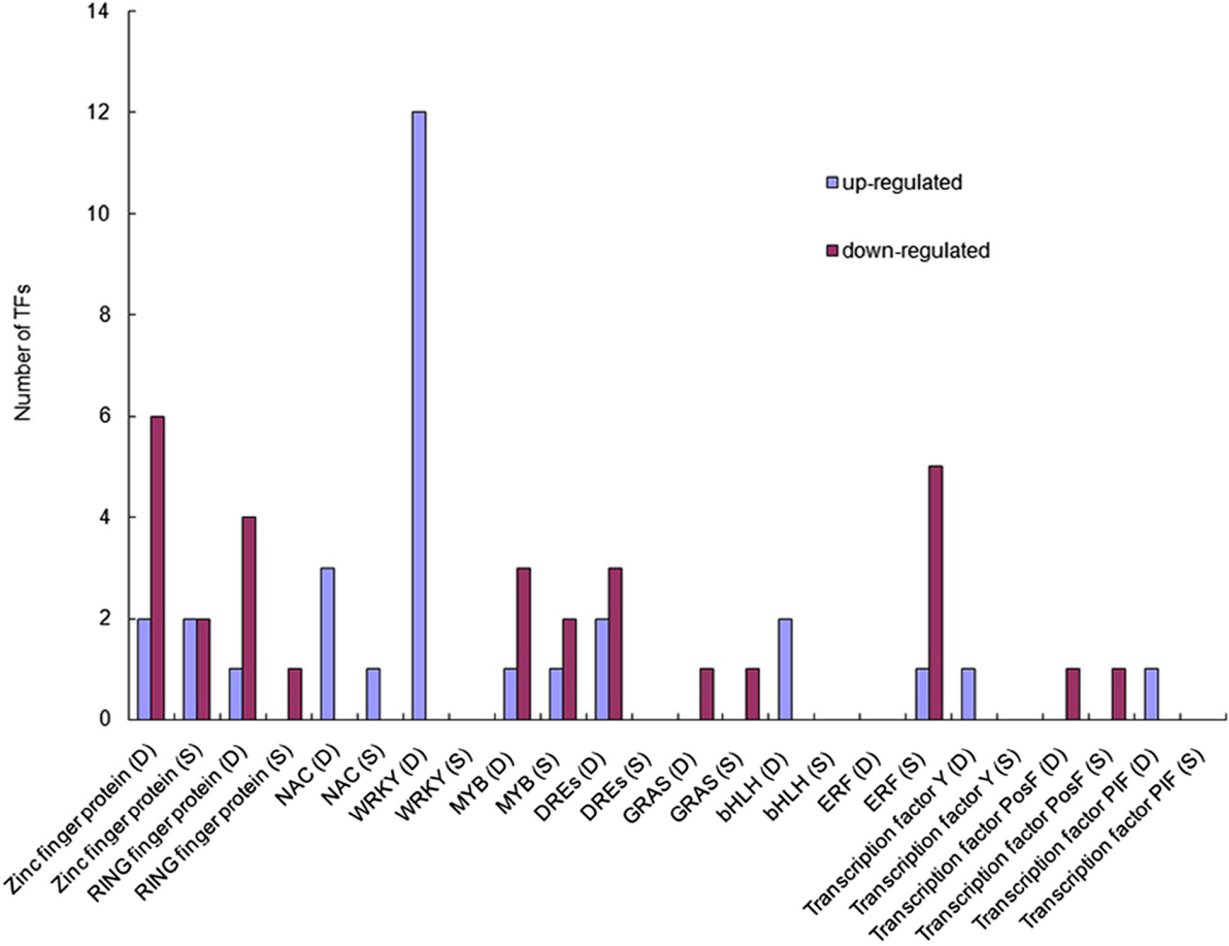
Differentially expressed transcription factors under drought or salt stress in *C. korshinskii*. D, transcription factors under drought stress; S, transcription factors under salt stress. *Blue bar* represents upregulated DEGs; *red bar* indicates downregulated DEGs

We performed global transcription-factor classification for differentially expressed transcripts and identified 53 transcripts belonging to 12 transcription-factor families (Fig. 8, Additional file 15). Several transcription-factor family genes, e.g., zinc-finger protein and NAC, were dramatically differentially expressed in both drought and salt treatments. In addition, 12 WRKY members were strongly upregulated under drought treatment. Among RING-finger proteins or MYB-family members, more were downregulated under drought treatment. Among DREs, the up- and downregulated members were evenly balanced.

### Genes of unknown function and genes unique to *C. korshinskii*

In this study, many contigs had no homologous matches among database sequences and thus have no known function. The vast set of un-annotated contigs (45.55 %, 58,960 of 129,451) could be novel genes not present in the known genomes and specific to *C. korshinskii* (Additional file 5). Among those unknown contigs, 124 (7.61 %) of 1630 DEGs and 114 (7.50 %) of 1521 DEGs were drought- or salt-responsive, respectively, in *C. korshinskii* (Additional file 7).

To identify genes that might be expressed uniquely in *C. korshinskii* under salt and/or drought stresses, we performed a new set of BLAST searches to compare these contigs to genes expressed in *Arabidopsis* under salt and/or drought stresses [37].

**Fig. 9:**
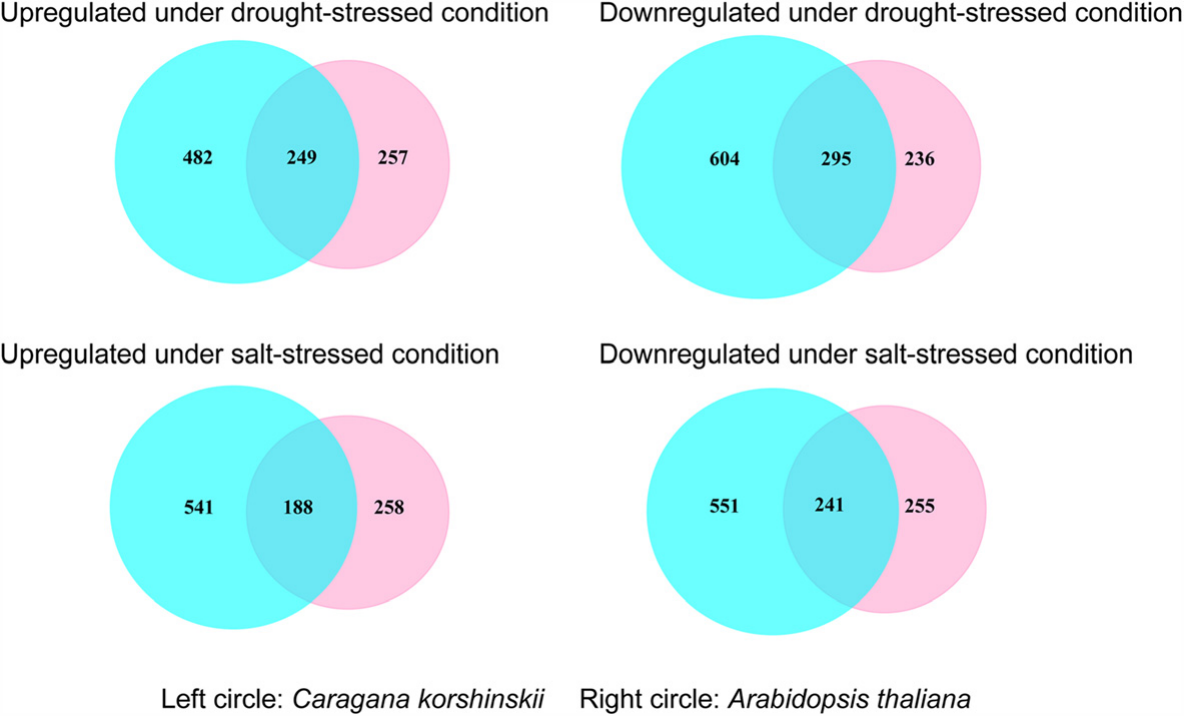
Venn diagrams illustrating the DEGs under drought or salt treatment in *C. korshinskii* and *Arabidopsis*. The *blue color* represents the upregulated or downregulated transcripts under drought or salt treatment in *C. korshinskii*, and the *red color* represent the upregulated or downregulated transcripts under drought or salt treatment in *Arabidopsis*, respectively

We found that 249 DEGs upregulated in drought-treated *C. korshinskii* were also upregulated in drought-treated *Arabidopsis* (Fig. 9, Additional file 16). These DEGs include some genes encoding cytochrome P450, glucosyltransferase, etc.; however, some genes (25 DEGs) that were strongly upregulated in drought-treated *C. korshinskii* did not significantly change in expression in drought-treated *Arabidopsis* (Additional file 16), such as 1-aminocyclopropane-1-carboxylate synthase and cysteine-rich receptor-like protein kinase. Many genes (227 DEGs) were significantly upregulated in drought-treated *C. korshinskii* and slightly downregulated in drought-treated *Arabidopsis* (Fig. 9, Additional file 16). Some of these genes encode flavin-containing monooxygenase, src2 protein, and similar proteins.

A total of 295 DEGs that were downregulated in drought-treated *C. korshinskii* were also determined to be downregulated in drought-treated *Arabidopsis* (Fig. 9, Additional file 16). Some of these genes encode nitrate reductase, glutathione S-transferase, etc.; however, some genes (29 DEGs) that were strongly downregulated in drought-treated *C. korshinskii* did not significantly change in expression in drought-treated *Arabidopsis* (Additional file 16), such as peroxidase and thiol protease. Many genes (249 DEGs) that were significantly downregulated in drought-treated *C. korshinskii* were upregulated in drought-treated *Arabidopsis* (Fig. 9, Additional file 16), including genes encoding subtilisin-like protease, ubiquitin carboxyl-terminal hydrolase, etc.

A total of 188 DEGs were upregulated in salt-treated *Arabidopsis* and *C. korshinskii*, such as spore-specific catalase and aldehyde dehydrogenase; however, many genes (17 DEGs) were strongly upregulated in salt-treated *C. korshinskii* but remained nearly constant in expression in salt-treated *Arabidopsis* (Fig. 9, Additional file 16), such as plasma membrane H^+^-ATPase and 60S ribosomal protein. Some genes (210 DEGs) that were significantly upregulated in salt-treated *C. korshinskii* were slightly downregulated in salt-treated *Arabidopsis* (Additional file 16), including genes encoding ADP, ATP-carrier protein, and mannitol dehydrogenase.

A total of 241 DEGs were downregulated in salt-treated *C. korshinskii* and *Arabidopsis* (Fig. 9, Additional file 16), including genes encoding LRR receptor-like serine/threonine-protein kinase and beta-glucosidase; however, some genes (33 DEGs) were strongly downregulated in salt-treated *C. korshinskii* but did not significantly change in expression in salt-treated *Arabidopsis* (Additional file 16), such as glutathione S-transferase and isoflavone conjugate-specific beta-glucosidase. Many genes (242 DEGs) that were significantly downregulated in salt-treated *C. korshinskii* were instead upregulated in salt-treated *Arabidopsis* (Additional file 16), including genes encoding peptide/nitrate transporter and E3 ubiquitin-protein ligase.

Of the 148 DEGs that were upregulated in both drought- and salt-treated *C. korshinskii*, 51 DEGs were also upregulated in both drought- and salt-treated *Arabidopsis* and 31 were downregulated in both drought- and salt-treated *Arabidopsis*. Of the 1630 DEGs upregulated in drought-treated *C. korshinskii*, 53 DEGs were upregulated and 35 DEGs were downregulated in drought-treated *Arabidopsis*. In salt-treated *Arabidopsis,* 53 of these DEGs were upregulated and 33 were downregulated. Six DEGs upregulated in drought- and salt-treated *C. korshinskii* remained relatively constant in both drought- and salt-treated *Arabidopsis* (Additional file 17).

Among 368 DEGs that were downregulated in both drought- and salt-treated *C. korshinskii*, 88 were upregulated in both drought- and salt-treated *Arabidopsis* and 117 were downregulated in both drought- and salt-treated *Arabidopsis*. Of the 1521 DEGs downregulated in drought-treated *C. korshinskii*, 95 were upregulated and 121 downregulated in drought-treated *Arabidopsis*, while, 93 of these DEGs were upregulated and 120 downregulated in salt-treated *Arabidopsis*. Twelve DEGs that were downregulated in both drought- and salt-treated *C. korshinskii* remained relatively constant in both drought- and salt-treated *Arabidopsis*, except that Cko_contig_45026 remained relatively constant in drought-treated *Arabidopsis* but not in salt-treated *Arabidopsis* (Additional file 17).

In drought-treated *C. korshinskii*, however, 542 DEGs (33.25 %) did not match any *Arabidopsis* sequences in this study and thus were presumably unique to *C. korshinskii*. The expression levels of 17 genes changed significantly in drought-treated *C. korshinskii*; for example, Cko_contig_61538 was predicted to encode E3 ubiquitin-protein ligase and was upregulated 790.9-fold (Additional file 18). In addition, genes for proteins such as phosphoinositide phospholipase C (Cko_contig_42646), trypsin-protein inhibitor (Cko_contig_23762, Cko_contig_27564), and bZIP transcription factor (Cko_contig_23833) were expressed uniquely in drought-treated *C. korshinskii*.

In addition, 529 DEGs (34.80 %) in salt-treated *C. korshinskii* had no homologous sequence in the *Arabidopsis* datasets and were considered unique to *C. korshinskii*. The expression levels of 27 genes changed dramatically in salt-treated *C. korshinskii*, such as Cko_contig_58120, predicted to encode polyphenol oxidase, which was upregulated 313.3-fold, and Cko_contig_92720, predicted to encode serine/threonine kinase, which was upregulated 2678.4-fold (Additional file 18). Other genes, such as thiol protease (Cko_contig_105443), UDP-glucosyltransferase (Cko_contig_3136), and polyphenol oxidase (Cko_contig_58120), were found to be uniquely expressed in salt-treated *C. korshinskii* (Additional file 18). Although we cannot yet be certain which genes are responsible for *C. korshinskii*’s stress resistance, we are convinced that the majority of predicted unigenes unique to *C. korshinskii* represent a valuable basis on which to explore gene diversity in *C. korshinskii* and support drought- or salt-stress resistance in *C. korshinskii*.

### Quantitative real-time-PCR validation of differentially expressed transcripts from RNA-seq

**Fig. 10:**
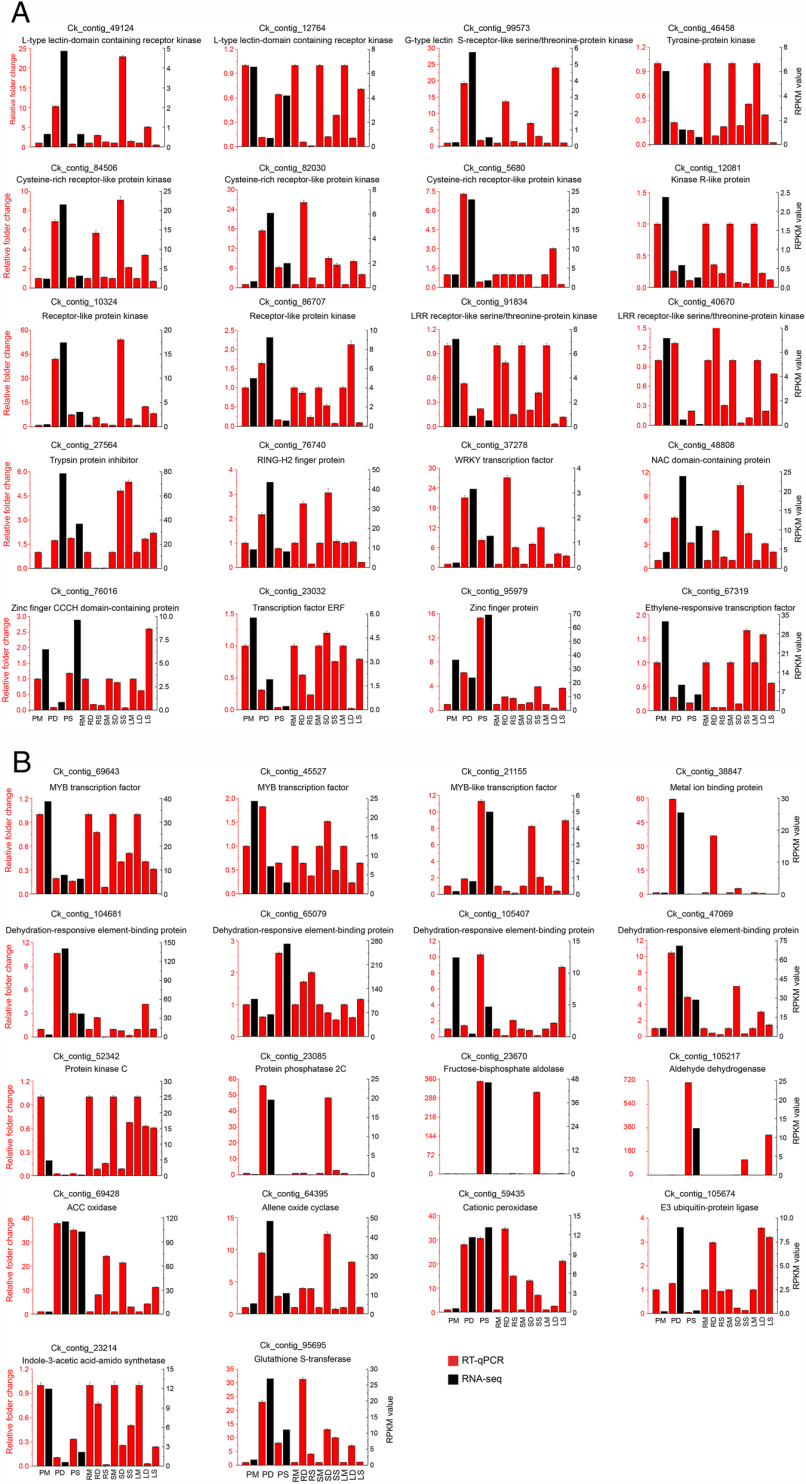
Changes in transcript levels of 38 selected genes, as detected by real-time RT-PCR. The *black bars* represent the relative intensity of qRT-PCR from three independent biological replicates (left Y-axis), and the *red bars* represent the expression level (RPKM) of the transcript (right Y-axis) (**a** and **b**). **a** includes 20 qRT-PCR results, and **b** includes another 18 qRT-PCR results. The qRT-PCR primers for each contig are listed in Additional file 19. PM, PD and PS represent that the tested materials were the whole seedlings including leaves, stems, and roots in normal growth condition, under drought treatment, and salt treatment, respectively. RM, RD and RS represents that the tested materials were the roots of the plant in normal growth condition, under drought treatment, and salt treatment, respectively. SM, SD and SS represent that the tested materials were the stems of the plant in normal growth condition, under drought treatment, and salt treatment, respectively. LM, LD and LS represent that the tested materials were the leaves of the plant in normal growth condition, under drought treatment, and salt treatment, respectively

To confirm the accuracy and reproducibility of the Illumina RNA-Seq results, a small number of contigs were chosen for quantitative real-time (qRT)-PCR detection. The expression of those contigs in root, stem, leaf samples of drought- or salt-treated *C. korshinskii* were analyzed (Fig. 10a and b). Detailed information on the primers is provided in Additional file 19. The qPCR results for 38 selected contigs showed general agreement with their transcript-abundance changes as determined by RNA-seq, suggesting the reliability of the transcriptomic profiling data. For example, under drought stress, Cko_contig_59435, which shows strong homology to cationic peroxidase 1, was upregulated 10.29-fold in *C. korshinskii*: 22.85-fold in the stem and 5.06-fold in the leaf (Fig. 10b). Cko_contig_12764, a homolog of L-type lectin-domain-containing receptor kinase, was upregulated 19.23-fold in *C. korshinskii* under drought stress: upregulated 13.68-fold in the root, 7.05-fold in the stem and 24.01-fold in the leaf (Fig. 10a).

Cko_contig_95695, a homolog of glutathione S-transferase, was upregulated 22.93-fold under drought stress and 8.08-fold under salt stress: 31.31-fold in the drought-treated root, 4.04-fold in the salt-treated root, 13.05-fold in the drought-treated stem, 10.03-fold in the salt-treated stem, 7.08-fold in the drought-treated leaf and 1.14-fold in the salt-treated leaf (Fig. 10b). Nonetheless, moderate discrepancies between the expression levels and RPKM values were observed for three contigs: Cko_contig_12764, Cko_contig_59435, and Cko_contig_27564 (Fig. 10a and b).

## Discussion

In this study, we generated *de novo* transcriptomes of *C. korshinskii* under drought and salt treatment and compared the expression profiles between control and drought and salt conditions, as well as between *C. korshinskii* and the model plant *Arabidopsis*. From our analysis, we found that many genes/contigs related to protein kinases, certain transcription factors, the ROS-scavenging system and ion transport proteins, gibberellin biosynthesis, carbon metabolism and osmotic regulation, and brassinosteroid-biosynthesis pathways are involved in drought- and salt-stress responses in *C. korshinskii*, with some of these genes being expressed significantly between *C. korshinskii* and *Arabidopsis*, as discussed in detail below. These results provide a molecular basis for understanding the specific drought- and salt-tolerance mechanisms of *C. korshinskii*.

### Construction of an informative transcriptome dataset for *C. korshinskii*

Transcriptome sequencing of *C. korshinskii* is fundamental to the identification of functional genes and the elucidation of the molecular mechanisms involved in drought or salt adaptation and tolerance in *C. korshinskii*. In a recent study in *C. korshinskii* [38], a total of 66.35 million raw sequencing reads were generated, 64.03 million clean reads were obtained, and 86,265 contigs were assembled. The percentage of annotation was 48.09 %. In our study, a total of 199.94 million raw reads were generated from the samples, and 182.93 million clean reads were assembled into 129,451 contigs (Table 1). Notably, the percentage of annotation was 54.45 %. The numbers of total raw reads, clean reads, assembled contigs and the percentage of annotation were all higher than those obtained previously [38], likely because we used several samples under different growth conditions for sequencing analysis, whereas only a sample under normal growth conditions was used by Long et al. [38]. Our *de novo* assembly of RNA-seq improves the genome annotation of *C. korshinskii*.

Based on comparisons with different plant species, the top four species with BLAST hits to annotated unigenes were *Glycine max*, *Medicago truncatula*, *Vitis vinifera*, and *Lotus japonicus*, species whose genome annotations are comprehensive and largely accepted. The low rate of annotation compared with other plants such as *Safflower Flowers* (58 %) [39], *Aegilops variabilis* (66.46 %) [40], and Ma Bamboo (78.9 %) [41] might be due to technological limitations, such as sequencing depth, splice junctions and read length [42], which are common in *de novo* assembly and RNA-seq analysis. We can speculate that the unannotated contigs (45.55 %, 58,960 of 129,451 contigs) represent a specific genetic resource for *C. korshinskii*, which is worthy of further investigation. Unannotated sequences were on average much shorter than the annotated contigs (334 bp vs 975 bp); however, a considerable percentage of contigs (20.2 %, or 14,209 of 70,491) that were longer than 500 bp and that had RPKM values [43] over three had no known homologs in known plant species.

### Protein kinases are important for drought and salt tolerance in *C. korshinskii*

Protein kinases phosphorylate and activate many target proteins in major signaling events in response to osmotic stress in higher plants [44]. Microarray analysis revealed that *Glycine soja* G-type lectin S-receptor-like serine/threonine-protein kinase *GsSRK* significantly increased in expression under alkaline (NaHCO_3_) treatment, identifying it as an alkaline-responsive gene [45]. In this work, Cko_contig_103451, associated with G-type lectin S-receptor-like serine/threonine-protein kinase, was upregulated 7.6-fold in drought-treated *C. korshinskii* whole plants and 4.6-fold in drought-treated *Arabidopsis* datasets. We also found that three other homologous genes (Cko_contig_99573, Cko_contig_39198, and Cko_contig_36276) were upregulated more than 32-fold in drought-treated *C. korshinskii* but changed little in *Arabidopsis* datasets (Additional file 20). These results are also consistent with the results of studies on the expression pattern of *GsSRK* in *Glycine soja* under NaCl (upregulated 4-fold in leaves and 3.5-fold in roots) and PEG treatments (upregulated 3.5-fold in leaves and 3.3-fold in roots) [46].

*Arabidopsis thaliana* L-type lectin-domain-containing receptor kinase *AtLPK1* is strongly induced by stress hormones and salt treatments, and overexpression of *AtLPK1* in *Arabidopsis* enhances salt-stress tolerance [39]. In this study, Cko_contig_12764 had a highly significant BLAST hit to the L-type lectin-domain-containing receptor kinase and showed increased transcript abundance in salt-treated *Arabidopsis* datasets (upregulated 3.5-fold), but was downregulated 8-fold in salt-treated *C. korshinskii*. Another homologous gene (Cko_contig_49124) was upregulated 7-fold in drought-treated *C. korshinskii* but upregulated slightly in *Arabidopsis* (Additional file 20). Kempa et al. [47] found that a plastid-localized glycogen synthase kinase 3 GSK-3 was induced at high concentrations of NaCl, KCl and sorbitol and could modulate salt-stress tolerance and regulate carbohydrate metabolism in *Medicago sativa* cv. Europa. We identified 58 contigs related to glycogen synthase kinase and only one differentially expressed transcript (Cko_contig_85930), which was uniquely expressed and upregulated 7.7-fold under drought stress in *C. korshinskii*, with no homologous gene detected in the examined *Arabidopsis* datasets.

In *C. korshinskii*, a large number of protein kinases may play an essential role in sensing external drought and salt signals and regulating gene expression at the cellular level in response, as seen in *Arabidopsis* (Additional file 20); however, the genes encoding protein kinases in response to stresses (e.g., salt or drought) may differ between *C. korshinskii* and other plants.

### Transcription factors involved in drought- and salt-stress response in *C. korshinskii*

Many WRKY, MYB, NAM, ATAF, and CUC (NAC) transcription factors were found to confer resistance to drought or salt stress in plants, and overexpression of these transcription factors enhanced the drought or salt tolerance of transgenic plants [7, 48–52]. Previous research has found that the expression levels of wheat (*Triticum aestivum* L.) *TaWRKY10* were upregulated 1.5-fold under PEG treatment and 2.6-fold under salt stress [53]. In the *C. korshinskii* and *Arabidopsis* datasets, we detected eight WRKY transcription factors that showed increased expression in both drought-treated *C. korshinskii* and *Arabidopsis* (Additional file 20), especially Cko_contig_52997 (upregulated 30.8-fold in drought-treated *C. korshinskii* and 12.3-fold in drought-treated *Arabidopsis*). The characteristics of WRKY expression in *C. korshinskii* and *Arabidopsis* were consistent with findings from research on wheat under drought stress [53]. The expression of the *Arabidopsis* zinc-finger proteins *Zat 11* and *Zat 12* is stimulated by drought (*Zat 12* was upregulated 10-fold in leaves) and salt stresses (*Zat 11* was upregulated 95-fold in roots) [54]. In this study, we found that a zinc finger protein, Cko_contig_79632, was upregulated 255.8-fold in drought-treated *C. korshinskii* but downregulated slightly in drought-treated *Arabidopsis,* and Cko_contig_85079 showed increased transcript abundance (20.0-fold increase) in salt-treated *C. korshinskii* but was only upregulated 0.9-fold in salt-treated *Arabidopsis*. Interestingly, we found that eight zinc-finger protein contigs were downregulated in drought- or salt-treated *C. korshinskii*. These findings support the possibility that zinc-finger proteins could play key roles as both positive and negative regulators of plant defenses [54].

We also identified differences in the expression profiles of some other transcription factors in *C. korshinskii* and *Arabidopsis* under the same stresses (Additional file 20); for example, some NAC, MYB, and ERFs were downregulated in drought- or salt-treated *C. korshinskii*. This result indicated that some MYB and ERF transcription factors might function as negative regulators of drought or salt tolerance in *C. korshinskii*.

### Functional genes related to the ROS-scavenging system and ion transport in *C. korshinskii*

ROS are produced in plant cells under normal and stressful conditions [55] and can be removed by the antioxidant system [55, 56]. In *C. korshinskii*, antioxidant enzymes such as superoxide dismutase (SOD), catalase (CAT), and peroxidase (POX) protect cells and subcellular systems from drought or salt damage caused by ROS (Additional file 20). For example, Cko_contig_105070 showed strong homology to CAT and was transcribed at high levels in salt-treated *C. korshinskii* (180.9-fold increase) and upregulated 1.13-fold in salt-treated *Arabidopsis* (Additional file 20). Therefore, it is most likely associated with enhanced CAT activity and responsible for converting the increased H_2_O_2_ into H_2_O and O_2_ [57]. Ascorbate peroxidase (APX) represents the ascorbate-dependent H_2_O_2_-scavenging mechanism in plants, which reduces H_2_O_2_ to H_2_O using ascorbic acid as an electron acceptor [57]. Eleven APX proteins were uniquely expressed in treated *C. korshinskii* (Additional file 5) but were not detected in *Arabidopsis* datasets. Two of these proteins (Cko_contig_33634, Cko_contig_46972) showed increased transcript abundance under drought stress. In the glutathione peroxidase (GPX) pathway, GPX can reduce H_2_O_2_ to the corresponding hydroxyl compounds [21, 58]. Interestingly, in our datasets, 12 GPX proteins were expressed uniquely in drought- or salt-treated *C. korshinskii*. In particular, Cko_contig_17138 was upregulated 2.4-fold in drought-treated *C. korshinskii* and upregulated 79.1 % under salt stress, no homologous contigs were found in published *Arabidopsis* datasets (Additional file 5).

It is possible that the survival of plants under salt stress depends on the accumulation of high levels of ions in the tissue for the maintenance of osmotic balance [59]. Cko_contig_24748, associated with a K^+^-transporter, was uniquely upregulated 16.5-fold under salt stress in *C. korshinskii* but was not found in the considered *Arabidopsis* datasets.

The transcript profile was consistent with the profile of K^+^-transporters in *R. trigyna*, most of which were upregulated under salt stress [21]. This result suggests that K^+^-transporters were enhanced and Na^+^/K^+^ homeostasis reestablished in salt-treated *C. korshinskii*, as seen in *R. trigyna* under salt stress [21]. It is possible that plasma-membrane H^+^-ATPases (PM-H^+^-ATPases) are involved in the mitigation of physiological disturbances imposed by salt stress [60]. Interestingly, in this study, Cko_contig_104930, encoding plasma membrane H^+^-ATPases (PM-H^+^-ATPases), showed significant increases in expression (3.1-fold increase) in salt-treated *C. korshinskii* but remained relatively constant in salt-treated *Arabidopsis*. This finding echoes the results of other studies on salt-treated plants, such as *Oryza sativa* Indica cultivars [60]. Transcripts encoding proteins associated with functions such as diterpenoid biosynthesis mechanosensitive ion channels (Cko_contig_66582) accumulated in both drought- and salt-treated *C. korshinskii* but were not detected in *Arabidopsis* datasets. These results indicate that *C. korshinskii* may have the specific ability to transduce these stress stimuli into electrical signals to enhance drought or salt tolerance. In summary, a small number of contigs uniquely found in *C. korshinskii* but not in *Arabidopsis* suggest that *C. korshinskii* may have a stress-tolerance mechanism distinct from that of the model plant *Arabidopsis*.

### Contigs in response to gibberellin biosynthesis in *C. korshinskii*

Plant growth and development are regulated by gibberellins (GAs), which are regulated by numerous genes involved in GA biosynthesis and degradation. GA biosynthesis is tightly controlled by Gibberellin 20-beta-dioxygenase (GA20ox) and Gibberellin 3-beta-dioxygenase (GA3ox). Gibberellin 2-beta-dioxygenase (GA2ox) catalyzes the deactivation of bioactive GAs [61]. In *Arabidopsis*, the cellular concentration of bioactive GAs was reduced via an increase in expression of GA2ox7, resulting in an accumulation of DELLA, an inhibitor of plant growth [62]. Based on transcriptome-sequence analysis of *C. korshinskii* and *Arabidopsis*, we identified nine drought- or salt-responsive GA2ox proteins, but these contigs were not found in *Arabidopsis* datasets (Additional file 5). Only five GA3ox proteins and Cko_contig_63577 were overexpressed in both-drought treated *C. korshinskii* and in researched *Arabidopsis* datasets (Additional file 20). Two GA20ox proteins were detected at higher levels in drought- or salt-treated *C. korshinskii*, but no homologous gene was found in the *Arabidopsis* datasets (Additional file 5). In addition, three contigs associated with DELLA protein were downregulated in both treated *C. korshinskii* and *Arabidopsis* (Additional file 20). Thus, we propose that GAs are upregulated under salt stress for normal growth of halophytes such as *C. korshinskii*, with accompanying repression of the expression of DELLA proteins. Our prediction is supported to some degree by studies of a GA-deficient mutant of *Arabidopsis* [62, 63].

### Contigs associated with carbon metabolism and osmotic regulation in *C. korshinskii* and *Arabidopsis*

Responses of carbon metabolism or starch metabolism to drought stress have been found in many plants, such as alfalfa (*Medicago sativa*) [64] and maize (*Zea mays*) [65]. In *C. korshinskii*, seven sugar-transport proteins, 13 carbohydrate-transport proteins, one granule-bound glycogen (starch) synthase protein and two sucrose-UDP glucosyltransferase proteins were found to be differentially expressed under drought or salt stress (Additional file 5). In particular, Cko_contig_43585, encoding a sugar-transport protein, was upregulated 568.5-fold in salt-treated samples. The expression data suggest that sugar or carbon metabolism, as well as various sugar-related signaling pathways, are influenced by drought stress in *C. korshinskii*.

The synthesis and transport of substances involved in osmotic regulation (such as proline and other amino acids) promote salt tolerance in most plants and crops [3, 66, 67]. Previous studies have reported that ProT accumulation is increased under salt stress in several plants, such as barley and mangrove [68, 69]. Our experimental results showed an increase in expression of the proline transporter gene (ProT, Cko_contig_90699), which was uniquely upregulated 2.6-fold and 1.7-fold in drought- and salt-treated *C. korshinskii,* respectively, but not in *Arabidopsis*. This drought- or salt-treated expression pattern in *C. korshinskii* was similar to the findings in barley and mangrove [68, 69]. In yeast, amino-acid permeases (AAPs) complement proline-uptake deficiency and mediate the efficient transport of proline, alanine, and valine, but the expression of most members of the AAP-gene family was repressed under high-salt conditions [70]. Surprisingly, the transcription of AAP (Cko_contig_4505) was uniquely induced (3.3-fold increase) in drought-treated *C. korshinskii*, and no homologous gene was found in published *Arabidopsis* datasets. This finding suggests that *C. korshinskii* had the distinct ability to synthesize and transport various osmoprotectants, such as proline, osmotin, and amino acids, to maintain cell turgor under osmotic stress, which is not seen in the model plant *Arabidopsis* or in other eukaryotes.

### Contigs associated with brassinosteroid-biosynthesis pathways in *C. korshinskii*

Brassinolides regulate plant growth and development and have been known to improve plant tolerance to abiotic stresses [2]. The results of the RNA-seq expression analysis showed that 30 contigs involved in brassinosteroid biosynthesis pathway were regulated in treated *C. korshinskii* (Additional file 5). Cko_contig_7817 (85A1/2 or 85A2), a homolog of brassinosteroid-6-oxidase family protein, was differentially regulated in treated *C. korshinskii* and *Arabidopsis* transcriptome datasets (Additional file 20, Additional file 21). We further found four contigs, Cko_contig_82841 (2.4.1.13), Cko_contig_8184 (3.2.1.26), Cko_contig_32733 (3.2.1.21), and Cko_contig_74975 (2.7.1.4), that are involved in starch and sucrose metabolism in drought- or salt-treated *C. korshinskii*, but no homologous genes were detected in *Arabidopsis* datasets (Additional file 5). These genes are necessary to enhance the biosynthesis of soluble sugars such as glucose, sucrose, and fructose and to maintain cell osmotic adjustment and balance (Additional files 22 and 23).

## Conclusions

This work presents an original transcriptome-sequencing analysis of mixed RNA from *C. korshinskii* whole seedlings (leaves, stems, and roots) under drought and salt stresses. A total of 129,451 cDNA contigs from whole *C. korshinskii* plants were obtained, of which 54.51 % were annotated with gene descriptions, while the remaining 45.49 % were unknown. A large number of candidate functional genes were identified as being potentially involved in multiple mechanisms that might contribute to drought or salt tolerance, meriting further investigation. Several key pathways, such as metabolic pathways, biosynthesis of secondary metabolites, ribosome, purine metabolism and plant hormone signal transduction, and GO terms, such as metabolic process, cell or cell part, and catalytic activity, were identified as being involved in drought and salt tolerance in *C. korshinskii*. In addition, hundreds of DEGs appeared to be expressed uniquely in *C. korshinskii* under drought and salt stress, respectively, compared to the published *Arabidopsis* database. These unknown genes and uniquely expressed DEGs were presumably genes unique to *C. korshinskii* that are involved in drought or salt tolerance. This information of *C. korshinskii* genes will be very useful in future studies on molecular adaptations to abiotic stress in leguminous plants and will facilitate the genetic manipulation of important plants.

## Methods

### Plant materials, growth conditions, and stress treatments

Seeds of *C. korshinskii* collected from Turpan Desert Botanical Garden, Chinese Academy of Sciences were sown in plastic pots (12 × 12 cm) filled with a nonsterile sandy soil irrigated with 1 L sterile water. After germination, *C. korshinskii* seedlings were grown in a greenhouse with a 16 h light/8 h dark photoperiod at a temperature of 22 °C ± 1 °C and a relative humidity of 50 ± 10 % and reirrigated with 0.7 L sterile water. Two weeks later, the salt-treated plants were watered with 400 mM NaCl, the drought-treated plants were unwatered, and control plants were irrigated with 1 L sterile water. One month later, when seedlings were approximately 10-cm high, all seedlings, leaves, stems, and roots were harvested for analysis.

### cDNA-library preparation and Solexa sequencing for transcriptome analysis

Total RNA was extracted from *C. korshinskii* whole seedlings treated with 400 mM NaCl or drought stress using a Plant RNA Extraction Kit (Autolab, China), following the manufacturer’s protocol. The concentration and quality of each RNA sample were determined using a NanoDrop 2000TM micro-volume spectrophotometer (Thermo Scientific, Waltham, MA, USA) and gel electrophoresis. The poly (A) mRNA was isolated from 10 mg total RNA using magnetic oligo(dT) beads, then divided into short fragments by fragmentation buffer (Ambion, Austin, TX, USA). The first-strand cDNA was synthesized by random-hexamer primers, and then the second-strand cDNA was synthesized using DNA polymerase I (New England Biolabs), RNase H (Invitrogen), buffer, and dNTPs. Short DNA fragments were purified with a QIAquick PCR Purification Kit (Qiagen Inc., Valencia, CA, USA), then subjected to end repair and poly(A) addition and ligated to sequencing adaptors. Suitable fragments (350–450 bp) were purified by agarose-gel electrophoresis and gathered by PCR amplification. One cDNA library (Control sample) was sequenced on a PE flow cell and three cDNA libraries (Control, drought-treated and salt-treated samples) were sequenced on a SE flow cell using the Illumina HiSeq™ 2000 platform.

### *De novo* transcriptome assembly

To facilitate assembly, the following criteria were used to filter low-quality reads:

1. Filter reads with adapter contamination.
2. Filter reads in which unknown nucleotides >5 %.
3. Filter reads in which more than 20 % of bases showed a Q-value less than 20.

High-quality clean reads obtained after the above filtering and correction steps were used for *de novo* assembly using the Trinity method [71] with an optimized k-mer length of 25 by default and with all other parameters set to default values. The assembled sequences were further incorporated into distinct contigs by using the CD-HIT [72] and CAP3 [73] programs. The parameters in CAP3 were “-p 98 -o 50”. CD-HIT was also used to cluster transcripts on the basis of sequence similarity (-c sequence identity threshold >0.98) and retain only the longest transcript from each cluster; redundant contigs were excluded.

### Sequence annotation

Multiple complementary approaches were adopted for alignment and analysis. All contig sequences were entered as search terms in the NCBI non-redundant nucleotide sequence (nr) database, the Kyoto Encyclopedia of Genes and Genomes (KEGG) database, the Cluster of Orthologous Groups of proteins (COG) database, and the Swiss-Prot protein database using a cut-off E-value of 10^−5^. Gene Ontology (GO) annotations of the contigs were determined using Blast2go (https://www.blast2go.com/) [74] according to the molecular function, biological process, and cellular component ontologies (http://www.geneontology.org/) with an E-value threshold of 10^−5^; the GO level of the GO distribution figure was three. The best alignment results were used to determine the 5′-to-3′ orientation of the contigs. Annotated contigs with no GO hits were investigated in terms of protein domains using InterProScan databases (http://www.ebi.ac.uk/interpro/). The “GetORF” program from the EMBOSS software package was used to predict the open reading frames (ORFs) of each contig [75]. Enzyme mapping of the annotated contigs was performed by direct GO-to-enzyme mapping and used to query the KEGG to define the KEGG orthologs (KOs). The KEGG mapping tool was used to plot these KOs into a complete metabolic atlas [76].

### Identification of differential expression transcripts (DEGs) and functional analysis

The expression of each contig was calculated by the RPKM method [43]. The method of Audic and Claverie [77] was applied to determine the *P*-value, which corresponds to the differential transcript expression of the contigs. The false discovery rate (FDR) is used to determine the *P*-value threshold in multiple tests. FDR ≤0.05 and the absolute value of log_2_Ratio ≥1 were used as the threshold to judge the significance of differential gene expression. The differentially expressed genes were used for GO- and KEGG-enrichment analyses. GO-enrichment analysis was performed using the Blast2go software, and the *P*-values were calculated using the Benjamini-Hochberg (BH) correction [78]. We selected a corrected *P*-value <0.05 as a threshold to determine which DEGs were significantly enriched in GO terms. In KEGG-enrichment analysis, the cellular metabolism, biochemical pathways, and potential biological behaviors of the differentially expressed genes were examined (www.genome.jp/kegg/). A corrected *P*-value <0.05 was used as the threshold to determine significant enrichment of the gene sets.

We performed a new set of BLAST searches to compare *C. korshinskii* contigs to genes expressed in *Arabidopsis* under salt and/or drought stresses, in order to identify genes that might be expressed uniquely in *C. korshinskii* under salt and/or drought stresses. The RNA-seq data of *Arabidopsis* can be found in ArrayExpress and the accession number was E-GEOD-48235.

### Real-time quantitative reverse-transcription polymerase chain reaction (qRT-PCR) for validation and expression-pattern analysis

Total RNA was extracted from whole seedlings and organs (leaves, stems, and roots) of *C. korshinskii* treated with 400 mM NaCl or drought stress using a Plant RNA Extraction Kit (Autolab, China) according to the manufacturer’s protocol. Reverse-transcription reactions were performed using Superscript II reverse transcriptase (Invitrogen, Grand Island, NY, USA) according to the manufacturer’s instructions and 1 μg of total RNA and oligo(dT) primers.

Primers for qRT-PCR were designed using the Primer Premier 5.0 software (Premier Biosoft Int., Palo Alto, CA, USA). The sequences of the specific primer sets are listed in Additional file 19. The qRT-PCR analysis was performed using an ABI Prism 7500 sequence detector (Applied Biosystems, Foster City, CA, USA) and the SYBR® Premix Ex TaqTM Kit (TaKaRa, Tokyo, Japan) to detect transcript abundance. The qRT-PCR reactions were performed in 20-μl volumes containing 2 μl first-strand cDNA, 200 nM of each primer, and 10 μl of the 1 × SYBR PCR mixture with the following cycling programs: denaturation at 95 °C for 30 s, then 40 cycles of denaturation at 95 °C for 3 s and annealing and extension at 60 °C for 30 s. The specificity of the amplifications of all products were verified by melting curve analysis, which was conducted at 95 °C for 15 s, 60 °C for 60 s, and 95 °C for 15 s. Three replicates were conducted in parallel, and the results were normalized to the expression level of the constitutive 18S rRNA gene. The relative quantitative method (ΔΔCt) was used to evaluate quantitative variation.

## Availability of data and materials

The RNA-seq data of *Caragana korshinskii* in this study was deposited into the National Center for Biotechnology Information (NCBI) Sequence Read Archive (SRA) database under number SRP061143.

The RNA-seq data of *Arabidopsis* that was used as reference data for the comparison between *Arabidopsis* and *C. korshinskii* can be found in ArrayExpress and the accession number was E-GEOD-48235.

## Additional files

Additional file 1: The data quality of RNA-seq. (XLS 34 kb) Additional file 2: Distribution of assembled contigs (A) and annotated contigs (B) in *C. korshinskii*. (TIF 776 kb) Additional file 3: Summary of sequence annotation for *C. korshinskii*. (XLS 24 kb) Additional file 4: Contigs that significantly matched the InterProScan database. (XLS 6568 kb) Additional file 5: Functional annotation of all contigs and RPKM expression values. The all-contigs set was used to search protein databases (nr, GO, Swiss-Prot, KEGG, COG, and InterProScan) using BLASTX (E-value <10^−5^). (XLS 14509 kb) Additional file 6: COG classification of putative proteins. A total of 94,820 tested contigs (73.15 %) from the all-contigs set were aligned to the COG database and classified into 25 functional categories. The Y-axis indicates the number of contigs in a specific functional cluster. (TIF 409 kb) Additional file 7: Functional annotation of DEGs and unknown DEGs in drought- and salt-treated *C. korshinskii*. Contigs with absolute values of |log_2_Ratio| ≥1 and FDR ≤0.05 were identified as DEGs. (XLS 830 kb) Additional file 8: GO categories of DEGs in drought- and salt-treated *C. korshinskii*. GO functional classification annotation provides a gene list and gene number for every specific GO term. (XLS 1124 kb) Additional file 9: GO distribution of upregulated or downregulated DEGs in drought- and salt-treated *C. korshinskii*. GO functional classification annotation provides a gene list and gene number for every specific GO term. (XLS 89 kb) Additional file 10: Summary of DEGs for drought- and salt-treated *C. korshinskii* enriched in KEGG pathways. The FDR values for all pathways are less than or equal to 0.05. (XLS 32 kb) Additional file 11: Summary of upregulated or downregulated DEGs in drought- and salt-treated *C. korshinskii* enriched in KEGG pathways. The FDR values for all pathways are less than or equal to 0.05. (XLS 30 kb) Additional file 12: Candidate functional genes involved in drought and salt tolerance in *C. korshinskii*. Among those functional genes, 171 contigs were upregulated and 170 contigs were downregulated in drought-treated *C. korshinskii*, while 100 contigs were upregulated and 189 contigs were downregulated in salt-treated *C. korshinskii*. (XLS 222 kb) Additional file 13: DEGs annotated to protein kinases in the data set. (XLS 82 kb) Additional file 14: Contigs associated with MAPK signaling pathways. (XLS 160 kb) Additional file 15: Differentially expressed transcription factors responded to drought and salt treatments in *C. korshinskii*. (XLS 28 kb) Additional file 16: Similarities and differences analysis of contigs between *C. korshinskii* and *Arabidopsis* datasets. (XLS 777 kb) Additional file 17: DEGs upregulated or downregulated in both drought- and salt-treated *C. korshinskii* expressed in *Arabidopsis* datasets. (XLS 211 kb) Additional file 18: DEGs expressed uniquely in *C. korshinskii* compared to *Arabidopsis*. (XLS 160 kb) Additional file 19: Primers of 38 contigs used to confirm the sequencing quality. (XLS 24 kb) Additional file 20: Related transcription factors and DEGs expressed in both *C. korshinskii* and *Arabidopsis*. (XLS 101 kb) Additional file 21: Summary of contigs related to plant brassinosteroid-biosynthesis pathways and their response to drought and salt treatment in *C. korshinskii*. Sequences presumed to have been captured in the library are shown in red. Contigs not captured in this library are presented in green. Cko_contig_7817 (85A1/2 or 85A2) showed high homology to brassinosteroid-6- oxidase family proteins and responded to drought (6.83-fold increase) and salt (8.04-fold increase) stresses in *C. korshinskii*. (TIF 16 kb) Additional file 22: Effects of drought and salt stresses on the expression of contigs associated with starch and sucrose metabolism. Sequences presumed to have been captured in the library are shown in red and deep green. *C. korshinskii* contigs not captured in this library are presented in pale green. Cko_contig_82841 (2.4.1.13), which most likely encodes sucrose synthase, was upregulated in both drought- and salt-treated *C. korshinskii*. (TIF 564 kb) Additional file 23: The expression of several contigs involved in starch and sucrose metabolism was enhanced in both drought- and salt-treated *C. korshinskii*. Sequences presumed to have been captured in the library are shown in red and deep green. *C. korshinskii* contigs not captured in this library are presented in pale green. Cko_contig_8184 (3.2.1.26), Cko_contig_32733 (3.2.1.21) and Cko_contig_74975 (2.7.1.4), which are involved in a-D-glucose or β-D-glucose biosynthesis, were upregulated in both drought- and salt-treated samples. (TIF 565 kb)
