# Supplementary figures and images for: Effects of drought and salt-stresses on gene expression in Caragana korshinskii seedlings revealed by RNA-seq
Source: BMC Genomics. 2016 Mar 8;17:200. doi: 10.1186/s12864-016-2562-0 (PMC4782325; doi:10.1186/s12864-016-2562-0)

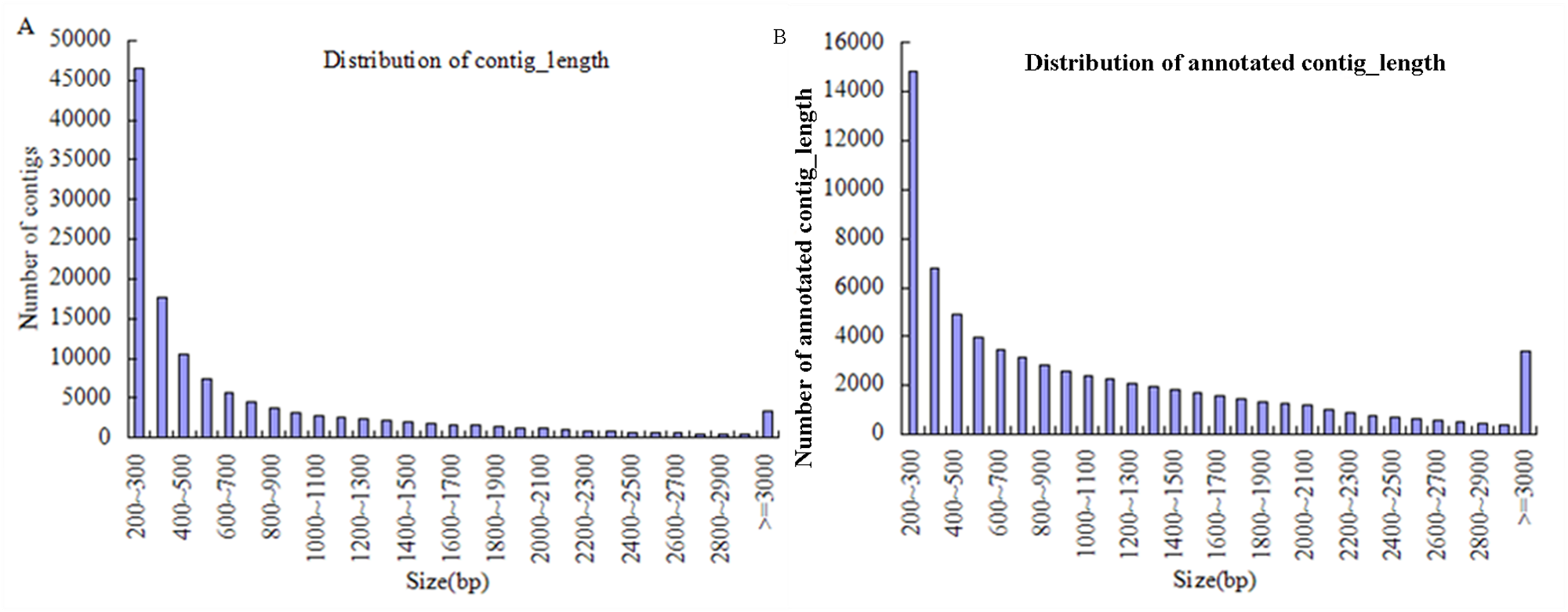

Supplement: Additional file 2: — Distribution of assembled contigs (A) and annotated contigs (B) in C. korshinskii. (TIF 776 kb) [file 12864_2016_2562_MOESM2_ESM.tif]

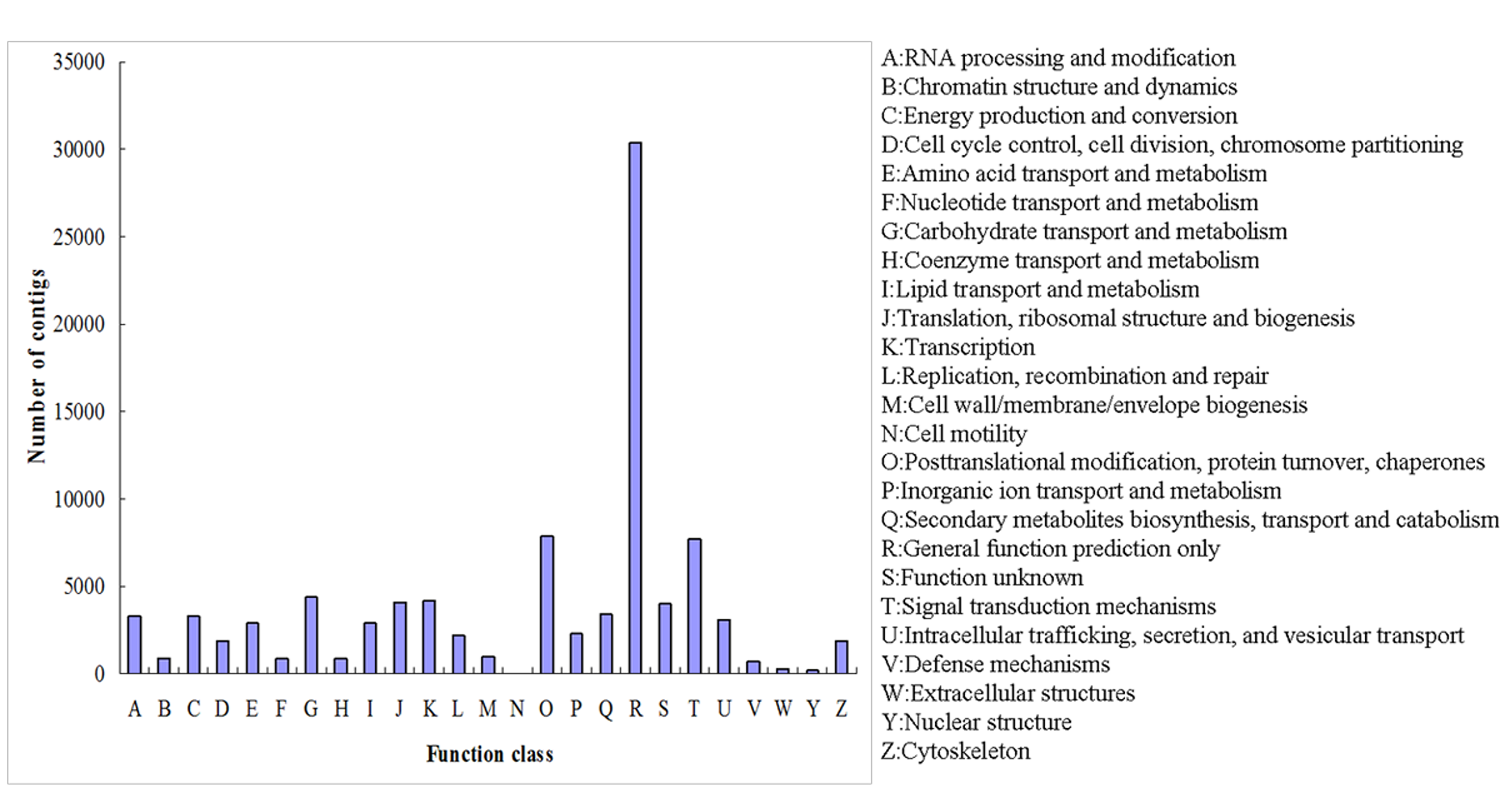

Supplement: Additional file 6: — COG classification of putative proteins. A total of 94,820 tested contigs (73.15 %) from the all-contigs set were aligned to the COG database and classified into 25 functional categories. The Y-axis indicates the number of contigs in a specific functional cluster. (TIF 409 kb) [file 12864_2016_2562_MOESM6_ESM.tif]

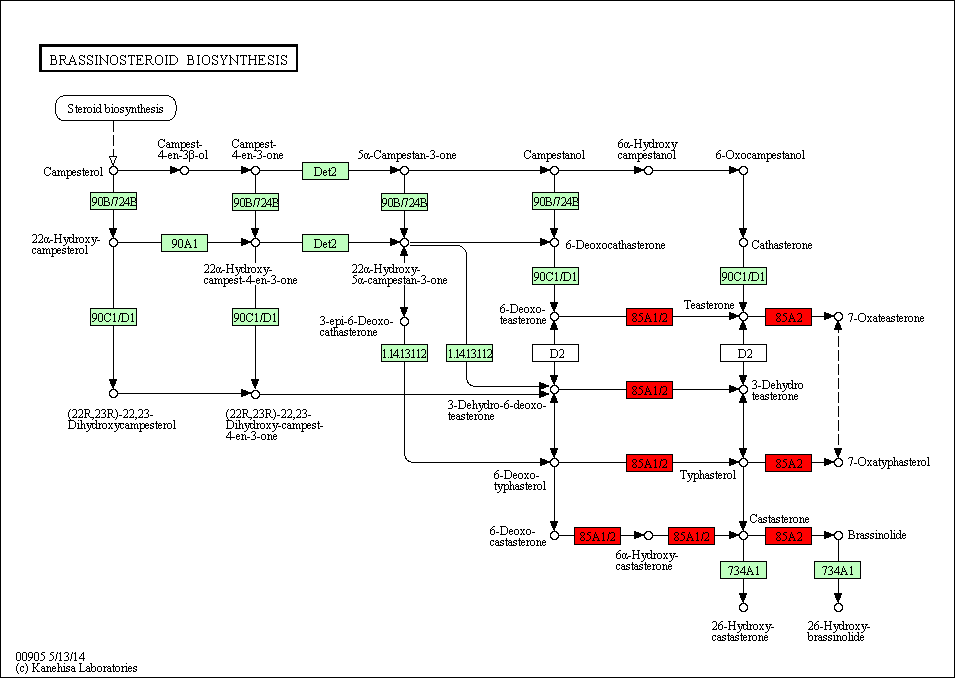

Supplement: Additional file 21: — Summary of contigs related to plant brassinosteroid-biosynthesis pathways and their response to drought and salt treatment in C. korshinskii. Sequences presumed to have been captured in the library are shown in red. Contigs not captured in this library are presented in green. Cko_contig_7817 (85A1/2 or 85A2) showed high homology to brassinosteroid-6- oxidase family proteins and responded to drought (6.83-fold increase) and salt (8.04-fold increase) stresses in C. korshinskii. (TIF 16 kb) [file 12864_2016_2562_MOESM21_ESM.tif]

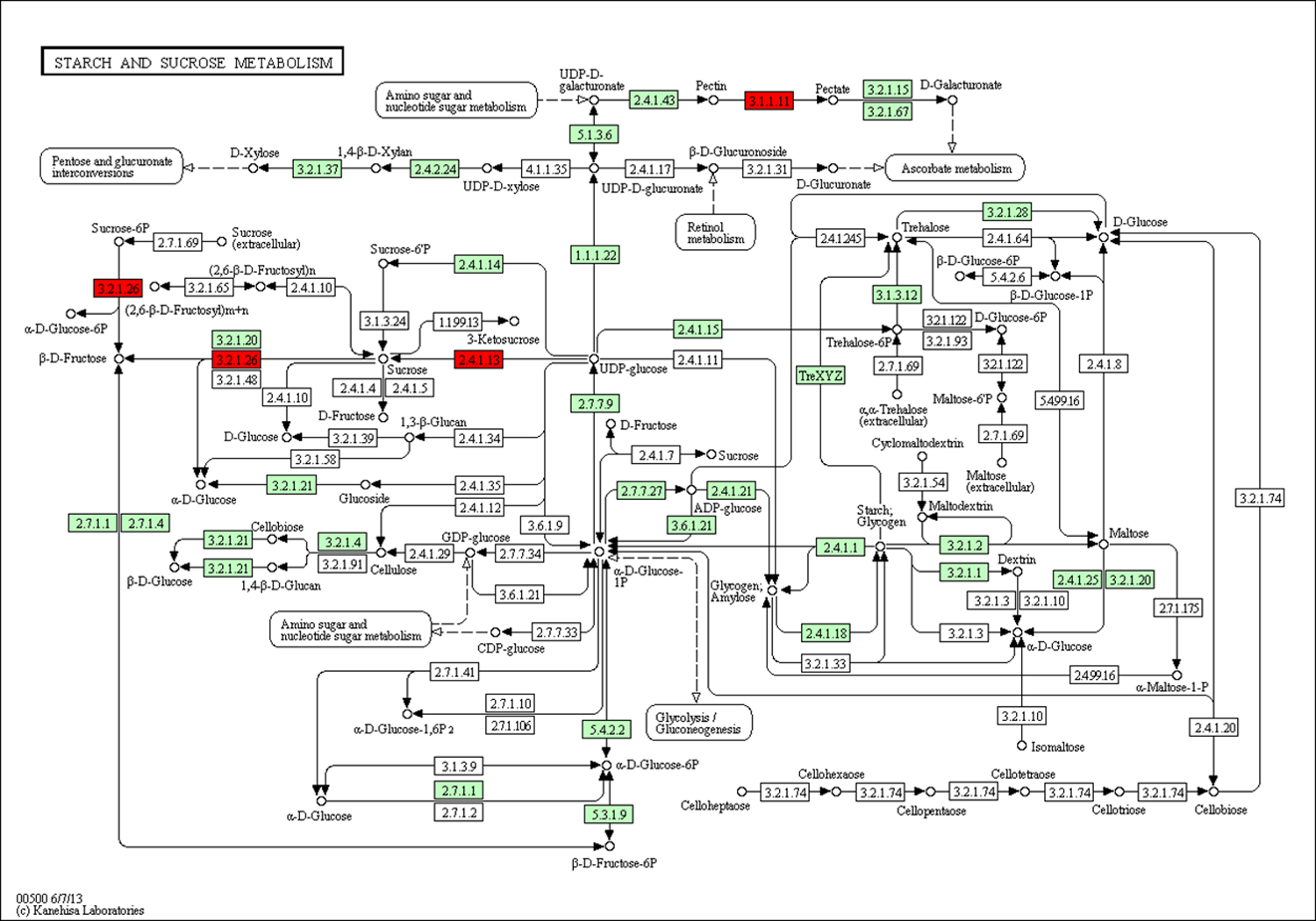

Supplement: Additional file 22: — Effects of drought and salt stresses on the expression of contigs associated with starch and sucrose metabolism. Sequences presumed to have been captured in the library are shown in red and deep green. C. korshinskii contigs not captured in this library are presented in pale green. Cko_contig_82841 (2.4.1.13), which most likely encodes sucrose synthase, was upregulated in both drought- and salt-treated C. korshinskii. (TIF 564 kb) [file 12864_2016_2562_MOESM22_ESM.tif]

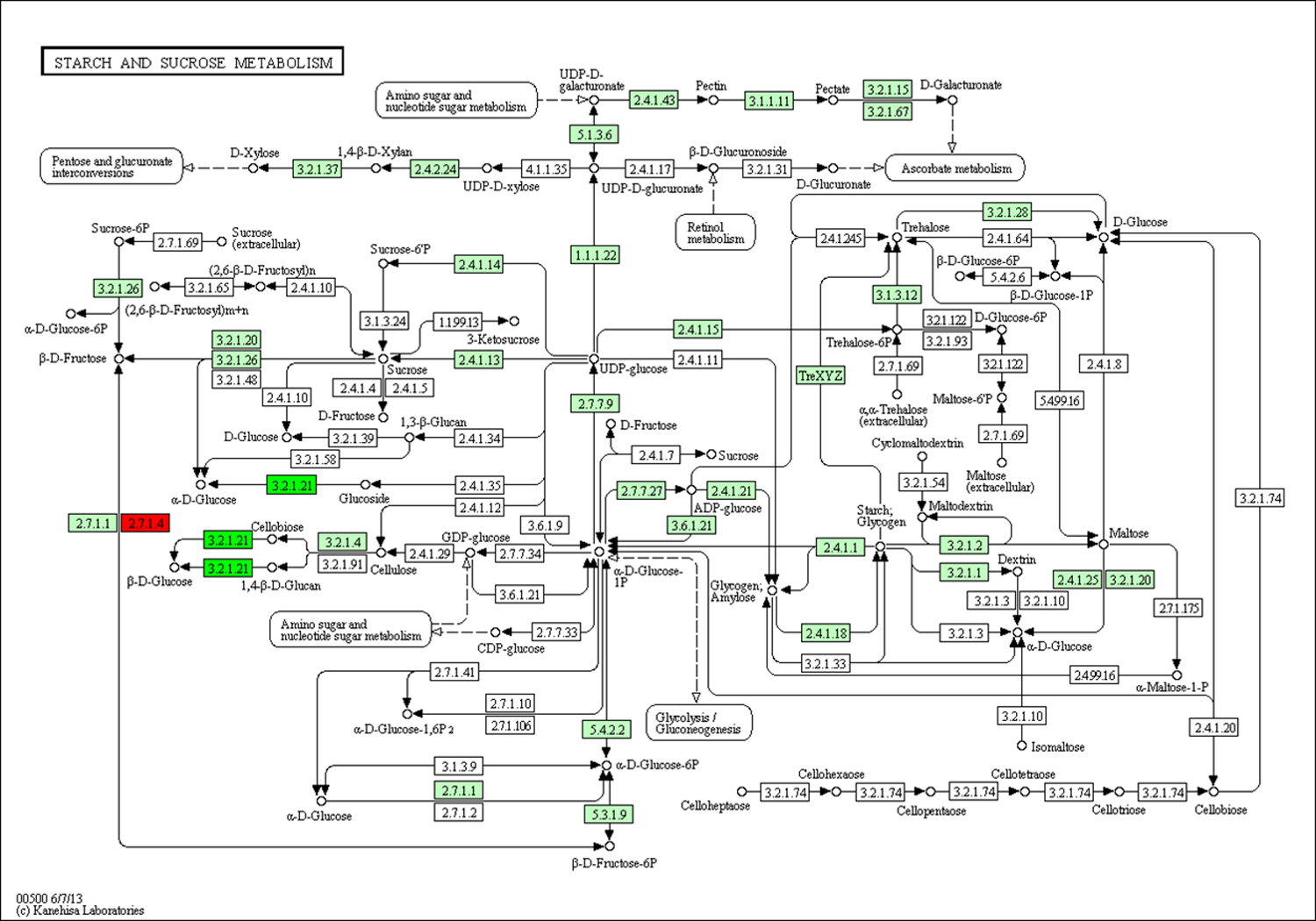

Supplement: Additional file 23: — The expression of several contigs involved in starch and sucrose metabolism was enhanced in both drought- and salt-treated C. korshinskii. Sequences presumed to have been captured in the library are shown in red and deep green. C. korshinskii contigs not captured in this library are presented in pale green. Cko_contig_8184 (3.2.1.26), Cko_contig_32733 (3.2.1.21) and Cko_contig_74975 (2.7.1.4), which are involved in a-D-glucose or β-D-glucose biosynthesis, were upregulated in both drought- and salt-treated samples. (TIF 565 kb) [file 12864_2016_2562_MOESM23_ESM.tif]
